# Pediatric and adult patients with ME/CFS following COVID-19: A structured approach to diagnosis using the Munich Berlin Symptom Questionnaire (MBSQ)

**DOI:** 10.1007/s00431-023-05351-z

**Published:** 2023-12-14

**Authors:** Laura-Carlotta Peo, Katharina Wiehler, Johannes Paulick, Katrin Gerrer, Ariane Leone, Anja Viereck, Matthias Haegele, Silvia Stojanov, Cordula Warlitz, Silvia Augustin, Martin Alberer, Daniel B. R. Hattesohl, Laura Froehlich, Carmen Scheibenbogen, Leonard A. Jason, Lorenz L. Mihatsch, Rafael Pricoco, Uta Behrends

**Affiliations:** 1 MRI Chronic Fatigue Center for Young People (MCFC), Pediatrics, Children’s Hospital, TUM School of Medicine, Technical University of Munich, Munich, Germany; 2 MRI Chronic Fatigue Center for Young People (MCFC), Child and Adolescent Psychsomatics, Children’s Hospital, TUM School of Medicine, Technical University of Munich, Munich, Germany; 3 German Association for ME/CFS, Hamburg, Germany; 4 Research Center CATALPA, FernUniversität in Hagen, Hagen, Germany; 5 Institute of Medical Immunology, Charité - Universitätsmedizin Berlin, Corporate Member of Freie Universität Berlin and Humboldt Universität zu Berlin and Berlin Institute of Health (BIH), Berlin, Germany; 6 Center for Community Research, DePaul University, Chicago, IL 60614 USA; 7 German Center for Infection Research (DZIF), Munich, Germany

**Keywords:** Children, Adolescents, ME/CFS, Post-COVID, SARS-CoV-2, Post-exertional malaise

## Abstract

A subset of patients with post-COVID-19 condition (PCC) fulfill the clinical criteria of myalgic encephalomyelitis/chronic fatigue syndrome (ME/CFS). To establish the diagnosis of ME/CFS for clinical and research purposes, comprehensive scores have to be evaluated. We developed the Munich Berlin Symptom Questionnaires (MBSQs) and supplementary scoring sheets (SSSs) to allow for a rapid evaluation of common ME/CFS case definitions. The MBSQs were applied to young patients with chronic fatigue and post-exertional malaise (PEM) who presented to the MRI Chronic Fatigue Center for Young People (MCFC). Trials were retrospectively registered (NCT05778006, NCT05638724). Using the MBSQs and SSSs, we report on ten patients aged 11 to 25 years diagnosed with ME/CFS after asymptomatic SARS-CoV-2 infection or mild to moderate COVID-19. Results from their MBSQs and from well-established patient-reported outcome measures indicated severe impairments of daily activities and health-related quality of life.

*   Conclusions:* ME/CFS can follow SARS-CoV-2 infection in patients younger than 18 years, rendering structured diagnostic approaches most relevant for pediatric PCC clinics. The MBSQs and SSSs represent novel diagnostic tools that can facilitate the diagnosis of ME/CFS in children, adolescents, and adults with PCC and other post-infection or post-vaccination syndromes.

**Table:** 

**What is Known:** *• ME/CFS is a debilitating disease with increasing prevalence due to COVID-19. For diagnosis, a differential diagnostic workup is required, including the evaluation of clinical ME/CFS criteria.* *• ME/CFS after COVID-19 has been reported in adults but not in pediatric patients younger than 19 years.*	
**What is New:** *• We present the novel Munich Berlin Symptom Questionnaires (MBSQs) as diagnostic tools to assess common ME/CFS case definitions in pediatric and adult patients with post-COVID-19 condition and beyond.* *• Using the MBSQs, we diagnosed ten patients aged 11 to 25 years with ME/CFS after asymptomatic SARS-CoV-2 infection or mild to moderate COVID-19.*

**Supplementary Information:**

The online version contains supplementary material available at 10.1007/s00431-023-05351-z.

## Introduction

The corona virus disease 2019 (COVID-19) pandemic has caused a global healthcare crisis. Besides immediate risks from severe acute respiratory coronavirus type 2 (SARS-CoV-2) infections [1, 2], post-acute sequelae of COVID-19 (PASC) are adding to the post-pandemic burden, straining healthcare and societies [3–7].

While many patients recover from PASC within few months, some endure a long lasting disorder, affecting social participation and health-related quality of life (HRQoL) [4, 8–10]. The World Health Organization (WHO) defined a post-COVID-19 condition (PCC) (ICD-10 U09.9!) as persistent or new symptoms 3 months after the initial SARS-CoV-2 infection (children: within 3 months), lasting over 2 months, and not explained otherwise [11, 12].

PASC affects at least 65 million individuals worldwide, with a population-based prevalence of about 10% of infected people and a lower prevalence in children [4, 13]. Estimating pediatric PCC prevalence is challenging [8, 14], with 0.8 to 13% reported in controlled cohorts [15, 16] and 2.0 to 3.5% calculated in a meta-analysis covering initially non-hospitalized, infected children and adolescents [17].

PASC/PCC may manifest with a wide variety of symptoms, including fatigue, shortness of breath, cognitive dysfunction, pain, sleep disorder, and/or mood symptoms. These symptoms can persist, fluctuate, or relapse and may have a significant impact on everyday functioning [11, 12, 18–20]. Some patients suffer from exertion intolerance with a worsening of symptoms after mild physical and/or mental activities, known as post-exertional malaise (PEM) [21, 22]. PEM can last for days or weeks and is recognized as a cardinal symptom of myalgic encephalomyelitis/chronic fatigue syndrome (ME/CFS) [23, 24]. ME/CFS following COVID-19 has been reported in adults [21, 25, 26] and in a 19-year-old male from the USA [27, 28] but, to our knowledge, not yet in younger patients. However, overlapping symptoms of PASC and ME/CFS have been described in pediatric patients [29].

ME/CFS is classified as a complex, chronic neurological disorder (ICD-10-GM G93.3 (Germany) or ICD-10-CM G93.32 (USA)), triggered mostly by infections [30–32]. Core symptoms include reduced daily functioning with fatigue not alleviated by rest, PEM usually lasting more than a day, unrefreshing sleep, neurocognitive deficits (“brain fog”) and/or orthostatic intolerance (OI), with additional symptoms in most cases [33]. Hypothesized pathogenic mechanisms of PCC and ME/CFS overlap, including viral persistence, latent virus reactivation, inflammation, autoimmunity, endothelial dysfunction, and microbiome dysbiosis [23, 34]. Common risk factors of PCC and ME/CFS are female gender, late adolescence or early adulthood, as well as pre-existing chronic health issues [14, 21, 31, 35, 36].

Population-based, pre-pandemic estimates of ME/CFS prevalence ranged from 0.1 to 0.89% in adults [37–40] and from 0.75 to 0.98% in adolescents and children [41, 42], with a high number of undetected cases [42]. Current estimates predicted at least a doubling of ME/CFS cases due to severe PCC [21, 27, 34, 43, 44].

Given the absence of a diagnostic ME/CFS biomarker, comprehensive evaluation is essential for complex disorders with chronic fatigue. Clinical criteria like the Canadian consensus criteria (CCC) [45] and the broader criteria by the former Institute of Medicine (IOM) [46] are widely used. For children and adolescents, the CCC were adapted by a “pediatric case definition” of L.A. Jason and colleagues (PCD-J) [47] and by the “clinical diagnostic worksheet” designed by P.C. Rowe and colleagues (CDW-R) [48]. A symptom duration of at least 6 months is usually required for adult patients [31], but was suggested to be reduced cross-age to facilitate early treatment [49]. For children and adolescents, the disease duration required by the CCC, the IOM criteria, and the PCD-J is 3 months [45–47].

ME/CFS care requires a holistic, longitudinal approach, including extensive patient education, the palliation of symptoms, and adequate psychosocial support. Patients must be carefully guided in “pacing” strategies to avoid PEM (“crashes”) [49].

Early identification of ME/CFS patients is important to prevent mismanagement and mitigate secondary harm, including disease deterioration and suicidality. Adequate care can lead to substantial improvement, particularly in young patients, often recovering within a decade [32]. However, recovery doesn’t imply absence of functional impairment [50]. Limited ME/CFS-specific awareness among healthcare providers [51–53], coupled with rising prevalence, increases the risk of inadequate care and secondary issues.

A challenge in clinical care and research for ME/CFS is the use of various diagnostic criteria and the lack of specific symptoms. To increase diagnostic sensitivity, the frequency and severity of symptoms should be assessed [54, 55].

The Munich Berlin Symptom Questionnaires (MBSQs) aim to facilitate the diagnostic approach across age goups with chronic fatigue following COVID-19 and beyond. They represent novel tools for an age-adapted, standardized evaluation of the most common clinical ME/CFS case definitions in clinical and research settings.

We introduce bilingual MBSQ versions and present results from the first ten PCC patients diagnosed with ME/CFS using the MBSQs in structured interviews at our MRI Chronic Fatigue Center For Young People (MCFC). Our Post-COVID clinic is part of the “Post-COVID Kids Bavaria” project, providing pediatric care and research for severe COVID-19 sequelae [56].

## Patients and methods

### Inclusion criteria and clinical assessment

Ten patients were diagnosed at the MCFC with PCC and ME/CFS using the German versions (can be requested from the authors) of the novel MBSQs and the supplementary scoring sheets (SSSs) (Supplementary information) (see description below). The collection and publication of medical data was approved by the TUM Ethics Committee (116/21, 511/21). Written informed consent was obtained from all participants (or parents) prior to inclusion. All patients had a history of confirmed (positive reverse transcription polymerase chain reaction (RT-PCR)) or probable (anti-SARS-CoV-2 IgG with a history of typical COVID-19 symptoms and without prior COVID-19 vaccination) SARS-CoV-2 infection and with post-viral symptoms lasting for more than 3 months.

Before visiting the MCFC, the patients completed various questionnaires in a stepped routine process, including well-established patient-reported outcome measures (PROMs) to assess fatigue (Fatigue Severity Scale (FSS) [57] or Chalder Fatigue Scale (CFQ) [58]), PEM (DePaul Symptom Questionnaire-PEM (DSQ-PEM)) [24], limitations in daily functioning (Bell Score) [59], HRQoL during the last 4 weeks (Short Form-36 Health Survey (SF-36)) [60], and the MBSQ.

Significant fatigue was indicated by a mean score of ≥ 5 (maximum: 7) in the FSS [61] or of ≥ 4 (maximum: 11) in the CFQ bimodal score [62]. The DSQ-PEM provides a Likert scale for the frequency (0–4) and severity (0–4) of five different PEM-related symptoms and evaluates the duration of PEM [24]. The Bell Score measures daily functioning on a scale from 0 to 100%, with 100% representing normal daily functioning [59]. The SF-36 consists of eight dimensions, including physical functioning, social functioning, vitality, general health, mental health, role physical, role emotional, and bodily pain. The score of each dimension is scaled to 0–100, with 0 representing the worst and 100 the best health status.

At the MCFC laboratory, technical tests were conducted to rule out other potential causes explaining the patients’ symptoms. Analyses varied based on symptoms, with core routine tests following prior recommendations [48]. Routine blood analyses included a differential cell count as well as C-reactive protein, liver, kidney, and thyroid function parameters, HbA1c, total serum immunoglobulins, antinuclear antibodies, antibodies against thyroid peroxidase, morning cortisol, antibodies against SARS-CoV-2 and Epstein-Barr virus (EBV), and EBV DNA load in blood and/or throat washes, supplemented by analyses of urine and stool (calprotectin, blood). Routine technical investigations included pulmonary function testing (PFT), electrocardiography, and ultrasound cardiography. If indicated, electroencephalography (EEG), cardiac or brain magnetic resonance tomography (MRT), ophthalmological, rheumatological, and/or other assessments were added.

In general, patients were jointly assessed by a pediatrician and psychologist or child and adolescent psychiatrist, specialized in ME/CFS. Alternatively, psychological evaluation was performed externally and reports discussed internally. All patients underwent a 10-min passive standing test to evaluate OI, including PoTS or orthostatic hypotension (OH) [63, 64]. The average HR while supine (5 min) was defined as baseline, and PoTS was defined by a sustained HR ≥ 120 beats per minute (bpm) and/or a sustained increase by HR ≥ 40 bpm for individuals ≤ 19 years and ≥ 30 bpm for individuals > 19 years in an upright posture (10 min), together with a history of orthostatic symptoms for at least 3 months [65, 66].

Clinical ME/CFS criteria were assessed through semi-structured interviews. MCFC physicians reviewed pre-filled MBSQs with patients (and parents) to prevent misunderstanding about symptoms and PEM duration. Home pre-filling saved time and let physicians focus on clarifications. ME/CFS diagnosis required at least one matched case definition and no other explanation of symptoms. An interdisciplinary ME/CFS board discussed each case involving experienced physicians.

### Development of the Munich Berlin Symptom Questionnaire

The MBSQ evaluates IOM and CCC criteria, which were recommended by the Centers for Disease Control and Prevention (CDC) [67] and the European Network for ME/CFS (EUROMENE) and require at least 6 months disease duration for adults (≥ 18 years) [31]. A separate version for children and adolescents (≤ 18 years) contains additional questions to assess the PCD-J and CDW-R criteria. For practicality, the pediatric MBSQ requires 3 months, though CDW-R advised preliminary diagnosis at 3 months and confirmed at six [48] (Table 1).

**Table 1:** MBSQ versions for different age groups

**Version**	**Adressed period** **of symptoms**	**Age group**	**CCC**	**IOM**	**PCD-J**	**CDW-R**
**Children and Adolescents**	Past 3 months	0–17 years	+	+	+	+
**Adults**	Past 6 months	≥ 18 years	+	+	−	−

*MBSQ *Munich Berlin Symptom Questionnaire, *CCC *Canadian Consensus Criteria, 2003 [45], *IOM *Criteria of the former Institute of Medicine, 2015 [46], *PCD-J *Pediatric Case Definition by Jason et al. [47], *CDW-R *Clinical Diagnostic Worksheet by Rowe et al. [48]

For developing the MBSQs, all terms used to describe the symptoms in the original publications [45–48] were mapped to the eight CCC symptom categories (fatigue, PEM, sleep disorder, pain, neurocognitive, autonomic, neuroendocrine, and immunologic manifestations). Overlaps and differences were identified, and umbrella terms introduced if neccessary. We aimed at the best match of all terms with terms in the original publications and adapted the wording, if necessary, during several rounds of clinical testing and discussion to optimize the understanding by patients and/or parents. The wording was not further adapted for children. The MBSQ was neither designed nor evaluated as a PROM and therefore is not recommended for use as such. The MBSQs are meant to aid a structured medical interview. This should exclude misunderstandings and may result in an adaptation of answers, if necessary. English versions are provided in the Supplementary information and German versions upon request from the authors.

We used a 5-point Likert scale for quantifying the frequency and severity of symptoms [54, 55]. In line with the DSQs, the MBSQs require an at least moderate frequency and severity (≥ 2) to support the ME/CFS diagnosis. Four additional dichotomous questions for the presence or absence of distinct features of fatigue or neurocognitive manifestations were included and three further questions for the prominent triggers of PEM, the main symptoms of PEM, and the most bothering symptoms of ME/CFS. In contrast to the DSQ-2 [55], the MBSQ focuses on ME/CFS symptoms only, omitting any further evaluation of medical history.

## Results

We developed the MBSQs and SSSs in German and English as novel tools for the clinical assessment of ME/CFS in the context of PCC and beyond. They address the most commonly recommended ME/CFS case definitions (CCC, IOM) and, in the versions for children and adolescents, two additional pediatric case definitions (CDW-R, PCD-J) (Table 1) to facilitate semi-structured, age-adapted approaches to diagnosis.

**Fig. 1:**
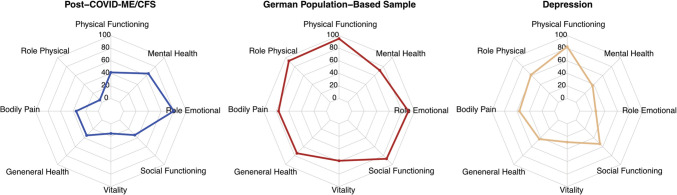
Results from the Short Form 36 Health Survey (SF-36). Spider diagrams display the different dimensions of the Short Form 36 Health Survey (SF-36) for the ten MCFC patients with ME/CFS following COVID-19 (Post-COVID-ME/CFS), the German norm population (age 14–20 years) from 1998 [68], and patients with moderate to severe depression (*n* = 60, mean age 17.5 ± 1.6 years) [70]

**Fig. 2:**
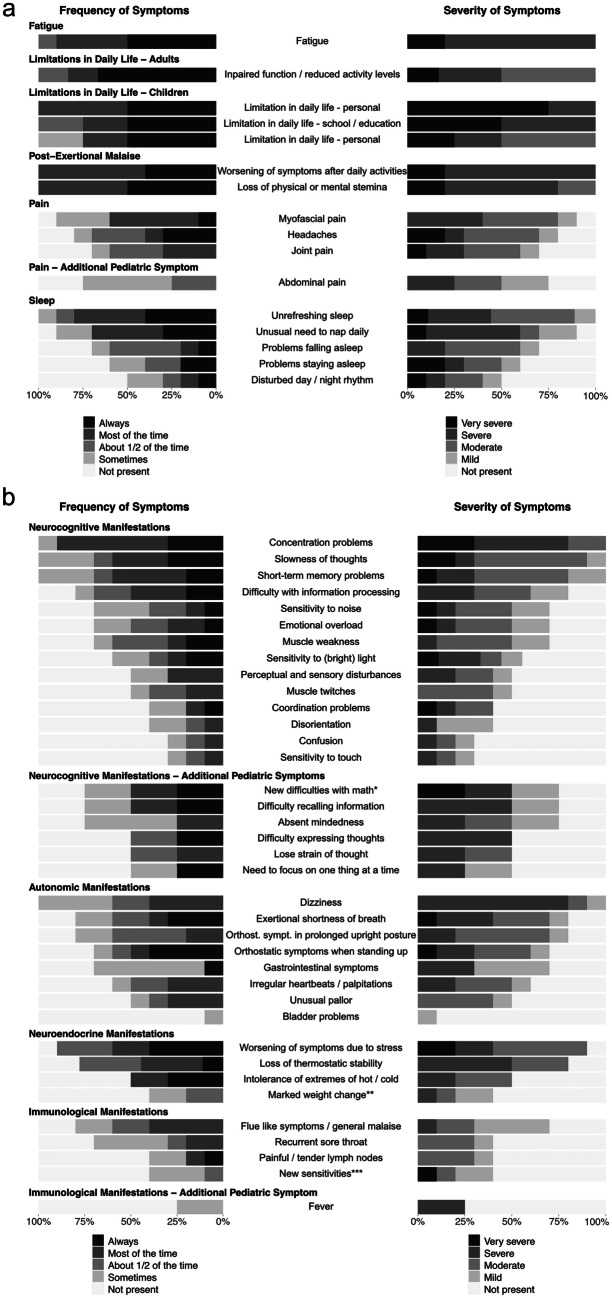
Frequency and severity of symptoms. **a** Stacked bar charts represent the frequency and severity of symptoms as indicated on the first page of the Munich Berlin Symptom Questionnaires (MBSQs). Symptoms that are assessed differently in pediatric (*n* = 4) and adult patients (*n* = 6) are presented separately, as indicated. **b** Stacked bar charts display the frequency and severity of symptoms from the second page of the Munich Berlin Symptom Questionnaires (MBSQs). Symptoms that are assessed differently in pediatric (*n* = 4) and adult patients (*n* = 6) are presented separately, as indicated. *New difficulties with math or other educational subject; **Marked weight change and/or loss of appetite and/or abnormal appetite; ***New sensitivities to food, medication or chemicals

Here, we applied the MBSQs to patients with PCC and report on the first ten patients diagnosed with ME/CFS after a thorough diagnostic workup (Tables 1 and 2, Figs. 1 and 2). Patients included an 11-year-old child, three adolescents (13 to 15 years), and six young adults (18 to 25 years). Three were males and seven females. At diagnosis, symptoms lasted 4 to 16 months (Table 2).

**Table 2:** Clinical data of patients with MECFS following SARS-CoV-2 infection

	**Patient**	**1**	**2**	**3**	**4**	**5**	**6**	**7**	**8**	**9**	**10**
	**Sex**	M	M	F	M	F	F	F	F	F	F
	**Age range (years)**	11–15				18–25					
**COVID-19**	**Loss of smell/taste**	−	−	+	−	−	+	+	−	−	+
	**RT-PCR**	+	+	n. d.	+	+	+	+	n. d.	+	+
	**Antibodies**^**a**^	+	+	+	n. d.	+	+	n. d.	+	n. d.	+
	**Medical care**	Non-hospitalized	Non-hospitalized	Non-hospitalized	Non-hospitalized	Non-hospitalized	Hospitalized	Non-hospitalized	Non-hospitalized	Non-hospitalized	Non-hospitalized
	**Latency period from infection to medical evaluation (months)**	10	14	16	4	10	10	13	14	11	9
**Post-COVID**	**Main symptoms** **as prioritized by the patient**	1. Dizziness	1. Concentration problems	1. Fatigue	1. Headaches	1. Concentration problems	1. Brain fog	1. Pain	1. Fatigue	1. Fatigue	1. Breathing problems
		2. Tiredness	2. Dizziness	2. PEM	2. Concentration problems	2. Fatigue	2. Headaches	2. Fatigue	2. Pain	2. Flu-like feeling	2. Malaise with mild fever
		3. Pain	3. Thermostatic instability	3. Neurocognitive manifestations	3. Dizziness	3. Headaches/ dizziness	3. PEM	3. Neurocognitive manifestations	3. Dizziness	3. PEM	3. Fatigue/ tiredness
	**OI/PoTS/OH**	OI	PoTS	OI	PoTS	OI	PoTS	PoTS	OI	-	PoTS
**PROMs**	**Bell Score**^**b**^	50–60	20	30	30	30	20–30	30	60	50	40–50
	**FSS**^**c**^	5.6	6.3	6.6	6.6	7.0	6.9	6.6	n. d	6.0	6.8
	**CFQ**^**d**^	n. d.	n. d.	n. d.	n. d.	n. d.	n. d.	n. d.	11	n. d.	n. d.
	**DSQ-PEM**	+	+	+	+	+	+	+	+	+	+
**MBSQs**	**CCC**	+	−	+	+	+	+	+	+	+	+
	**IOM**	−	+	+	+	+	+	+	+	−	+
	**PCD-J**	−	−	+	+	n. d.	n. d.	n. d.	n. d.	n. d.	n. d.
	**CDW-R**	+	+	+	+	n. d.	n. d.	n. d.	n. d.	n. d.	n. d.
	**PEM duration (hours)**	> 24	> 24	> 24	> 24	> 24	> 24	> 24	> 24	> 24	> 24

*M* male, *F *female, *RT-PCR *reverse transcription polymerase chain reaction, *n. d. *not done, *PEM *post-exertional malaise, *PoTS *postural orthostatic tachycardia syndrome, *OI* orthostatic intolerance, *OH *orthostatic hypotension, *PROMs *patient-reported outcome measures, *FSS *Fatigue Severity Scale, mean score, *CFQ *Chalder Fatigue Scale, bimodal score, *DSQ-PEM *DePaul Symptom Questionnaire-Post-Exertional Malaise, *MBSQs *Munich Berlin Symptom Questionnaires, *CCC *Canadian Consensus Criteria [45], *IOM *Criteria of the former Institute of Medicine [46], *PCD-J *Pediatric Case Definition by Jason et al. [47], *CDW-R *Clinical Diagnostic Worksheet by Rowe et al. [48], *h *hours

^a^anti-SARS-CoV-2 spike antibodies before vaccination and/or anti-SARS-CoV-2 nucleocapsid antibodies

^b^Bell Score (%) (0% = entirely bedridden; 100% = normal daily functioning)

^c^FSS (maximum value: 7)

^d^CFQ (maximum value: 11)

Nine of ten patients were diagnosed with confirmed or probable COVID-19, and one with asymptomatic SARS-CoV-2 infection from March 2020 to January 2022. Eight patients provided positive SARS-CoV-2 RT-PCR results, and two showed SARS-CoV-2 IgG antibodies with a history of COVID-19-like symptoms and no prior vaccination. One adult was hospitalized for a pre-syncopal episode in the context of COVID-19. One adolescent and three adults reported an initial loss of smell/taste (Table 2).

Pre-existing medical conditions were present in 9/10 patients, including bronchial asthma (4/10), hypothyroidism (2/10), Grave’s disease with hyperthyroidism (1/10), allergies (2/10), attention deficit disorder (1/10), migraine (1/10), history of meningitis (1/10), or Alport’s syndrome (1/10).

Differential diagnostics did not reveal any alternative causes for the debilitating symptoms. An adult patient’s cardiac MRT indicated prior perimyocarditis. Two patients showed bronchial hyper-responsiveness via PFT, with one reporting on pre-existing asthma. Neurologists recommended cranial MRT for nine and EEG for eight patients. Outcomes were mostly normal, except a stable, benign CNS lesion and a transient theta wave slowing in one patient. 9/10 patients complained of OI, with 5/10 patients meeting the diagnostic criteria for PoTS.

All patients showed significant fatigue based on FSS (9/9) or CFQ (1/1) and positive PEM based on the DSQ-PEM, with PEM duration ≥ 24 h (Table 2). Daily function (Bell Score) varied from 20 to 60% (median: 30, IQR: 30–48.75).

In SF-36 results (Fig. 1), all dimensions were impaired compared to German norms for ages 14 to 20 [68]. The physical component summary (PCS) was notably lower in our ME/CFS group (24.9 vs 53.4, *P* < 0.001). Mean mental health component summary (MCS) score was 44.9 vs 45.0 (*P* = 0.982) [68].

All patients experienced substantial reductions in occupational, educational, and/or personal activities, indicated by scoring at or below at least two of the three following subscale cut-offs on the SF-36: role physical ≤ 50, social functioning ≤ 62.5, and vitality ≤ 35, as required by the original CCC and the PCD-J [69]. In contrast to moderate to severe depression patients (*n* = 60, mean age 17.5 ± 1.6 years) [70], our ME/CFS patients scored notably lower in physical functioning (*P* < 0.001), role physical (*P* < 0.001), bodily pain (*P* < 0.001), vitality (*P* = 0.002), and social functioning (*P* = 0.016). They scored higher in role emotional (*P* < 0.001) and mental health (*P* < 0.001) compared to adolescents and young adults with moderate to severe depression. General health scores showed no significant difference (*P* = 0.082) (Fig. 1).

All patients fulfilled at least one ME/CFS case definition addressed in the MBSQ. One child fulfilled the CCC and the PDW-R but not the IOM and the PCD-J. Two adolescents met all four sets of criteria, while one met only the broader PDW-R and IOM criteria. All adults fulfilled the CCC, but one did not match the IOM criteria since sleep was not recognized as “unrefreshing” (Table 2).

Most common ME/CFS symptoms were fatigue (10/10), limitations in daily life (10/10), PEM (10/10), unrefreshing sleep (9/10), neurocognitive manifestations (10/10) (e.g., concentration and memory problems), and dizziness (6/10). The most bothering symptoms, the number, frequency, and severity of symptoms varied individually (Table 2, Fig. 2).

## Discussion

The MBSQs and SSSs are novel, age-adapted, concise diagnostic tools developed to facilitate the evaluation of ME/CFS criteria in patients with fatigue following COVID-19 and beyond. We reported ten young PCC patients who were diagnosed with ME/CFS using the MBSQs. To our knowledge, this is the first report on ME/CFS in people with PCC ≤ 18 years.

Limited data exists on severe PCC prevalence in children and adolescents. A survey ending on March 30, 2023, in the UK on self-reported PASC showed fewer children aged 2–11 (0.1%) with “limiting day-to-day activities” compared to older groups (12–24 years: 0.26–0.33%, ≥ 25 years: 0.46–0.92%) [71]. Early in the pandemic, a report from Sweden indicated long-term deficits in social participation due to pediatric PCC [72], and a single 19-year-old with post-COVID-ME/CFS was documented in the USA [27, 28]. Meanwhile, additional pediatric patients were diagnosed with ME/CFS at our MCFC and at pediatric partner sites of our multicenter registries (NCT05638724, NCT05778006), as will be reported in more detail (unpublished results).

ME/CFS after viral or other triggers is well documented in children and adolescents [48]. In a pre-pandemic pediatric cohort from Australia, ME/CFS was reported to have followed infections in up to 80% of cases, with EBV infection accounting for 40% of cases [32]. 12.9%, 7.3%, and 4.3% of adolescents in a pre-pandemic US cohort presented with ME/CFS as defined by the PCD-J criteria at six, 12, and 24 months after EBV-induced infectious mononucleosis [73]. ME/CFS defined by meeting at least one of three case definitions (Fukuda, IOM, CCC) manifested in 23% of US college students following symptomatic primary EBV infection, with 8% of the cohort fulfilling the CCC [74]. Pediatric ME/CFS showed recovery rates of 38% at 5 years and 68% at 10 years in Australia [32]. We recently published a first German cohort of adolescence with ME/CFS following EBV with partial recovery over time [75]. Pediatric ME/CFS post-SARS-CoV-2 was thus not unexpected and might be transient with proper diagnosis and treatment.

However, ME/CFS after SARS-CoV-2 infection was already documented in adults [21, 25, 27], but not yet in children. To our knowledge, this is the first report on ME/CFS in PCC patients aged ≤ 18 years, including a child as young as 11 years. All patients experienced persistent symptoms after asymptomatic SARS-CoV-2 infection or mild/moderate COVID-19, with no other explanation, aligning with WHO’s PCC definition [11]. A substantial reduction in occupational, educational, and/or personal activities of these patients was confirmed by a Bell score of ≤ 60% and by scoring at or below at least two of three subscale cut-offs on the SF-36 (role physical ≤ 50, social functioning ≤ 62.5, and vitality ≤ 35) [69].

Consistent with lower rates of severe PCC in children compared to older groups [71], 9/10 of our patients were older than 12 years. At the MCFC, we are regularly seeing young adults up to the age of 20 years, as their healthcare needs align with those of older adolescents. Exclusion of 18-year-olds from pediatric PCC studies might lead to data gaps [76].

Limited data on ME/CFS, in general, may partially result from insufficient disease-specific knowledge and experience [48, 51], and different ME/CFS case definitions render the comparison of published data challenging [77, 78]. Moreover, high time and cost expenses for the diagnostic workup may prevent clinicians from diagnosing ME/CFS and as a result these patients often get no adequate care. Thus, harmonization of diagnostic criteria for ME/CFS and concise diagnostic tools are urgently needed.

Our MBSQ approach was based on the DSQs developed as PROMs by L.A. Jason and colleagues to evaluate ME/CFS diagnosis and associated features in studies with adults, adolescents, and children [54]. Like the DSQs, the MBSQs offer Likert scales for the quantification of symptoms, with a threshold of ≥ 2 for both frequency and severity to indicate diagnostic relevance. Moreover, as introduced by the DSQs, the SSSs provide an algorithm to evaluate different case definitions using a single questionnaire.

Unlike the DSQ-2, the MBSQs do not address the international consensus criteria since they were not recommended by the EUROMENE [31].

Importantly, the MBSQs set a ≥ 14 h PEM duration cutoff for the CCC, PCD-J and CDW-R, but not the IOM criteria. Prior studies showed that most ME/CFS patients experience PEM lasting ≥ 24 h [24]. Some ME/CFS case definitions required a > 24 h duration, including the CDW-R (“Recovery takes more than 24h”) [48]. The original publications of the CCC and the PCD-J stated that “there is a pathologically slow recovery period–usually 24 h or longer” (CCC) [45] and that “the recovery is slow, often taking 24 h or longer” (PCD-J) [47]), respectively. We set the MBSQs’ PEM duration cut-off at ≥ 14 h, encompassing more ME/CFS patients than ≥ 24 h, while still differentiating from most other chronic diseases [24].

The MBSQs also incorporated the broader IOM criteria, endorsed by EUROMENE and CDC. The IOM criteria lack any requirements for PEM duration. Yet, assessing PEM duration in PCS is important as it defines subgroups of patients with different biomarker profiles and clinical courses [21, 79–82]. Consistent case definition use would enhance global ME/CFS healthcare data comparability, including PCC-related ME/CFS.

The MBSQs have limitations. Firstly, they haven’t been compared to other questionnaires, since MCFC patients already face multiple questionnaire challenges. However, the MBSQs are only suggested to aid structured medical interviews, a gold standard for diagnosing ME/CFS and not to be used as PROMs. Future goals include comparing the results of pre-filled MBSQs with results from medical visits and from other questionnaires. Secondly, due to limited cases’ diversity, correlations and consistency couldn’t be analyzed. This will be explored with a larger patient group. Thirdly, a structured interview based on the MBSQ can’t replace a future biomarker. Without the latter, diagnosing PEM, especially in successful pacers, remains tough. Lastly, we only discuss ME/CFS cases here, while ongoing research is evaluating the MBSQs in healthy individuals and other chronic diseases.

Taken together, the MBSQs and SSSs were developed to standardize and accelerate ME/CFS diagnosis at any age in clinical practice and research. They were successfully applied here to children, adolescents, and young adults with PCC. Standardization in PCC and ME/CFS research is urgently needed to compare clinical studies, identify biomarkers, and eventually select and develop specific treatment approaches [83].

## Supplementary Information

Below is the link to the electronic supplementary material.

Supplementary file1 (PDF 1.05 MB)

Supplementary file2 (PDF 1.10 MB)

## Data Availability

The data that support the findings of this study are available from the corresponding author, U.B., upon reasonable request.

## Abbreviations

bpm: Beats per minute
CCC: Canadian consensus criteria
CDC: Centers for Disease Control and Prevention
CDW-R: Clinical diagnostic worksheet of P.C. Rowe et al. [48]
CFC: Charité Fatigue Center
COVID-19: Coronavirus disease 2019
DSQ: DePaul symptom questionnaire
EBV: Epstein-Barr virus
EEG: Electroencephalography
EUROMENE: European Network on ME/CFS
GET: Graded exercise therapy
HR: Heart rate
HRQoL: Health-related quality of life
IOM: Institute of Medicine
MBSQ: Munich Berlin Symptom Questionnaire
MCFC: MRI Chronic Fatigue Center for Young People
MCS: Mental health component summary score
ME/CFS: Myalgic encephalomyelitis/chronic fatigue syndrome
MRI: TUM university hospital (Klinikum rechts der Isar)
MRT: Magnetic resonance tomography
NICE: National Institute for Health and Care Excellence
OH: Orthosthatic hypotension
OI: Orthosthatic intolerance
PASC: Post-acute sequelae of COVID-19
PCC: Post-COVID-19 condition
PCD-J: Pediatric case definition by L.A. Jason et al. [47]
PCS: Physical component summary score
PEM: Post-exertional malaise
PFT: Pulmonary function testing
PoTS: Postural tachycardia syndrome
PROM: Patient-reported outcome measure
RT-PCR: Reverse transcriptase-polymerase chain reaction
SARS-CoV-2: Severe acute respiratory coronavirus type 2
SEID: Systemic exertion intolerance disease criteria
SF-36: Short Form-36 Health Survey
SSS: Supplementary Scoring Sheet
UCG: Ultrasound cardiography
WHO: World Health Organization
