# Correction to: Pediatric and adult patients with ME/CFS following COVID-19: A structured approach to diagnosis using the Munich Berlin Symptom Questionnaire (MBSQ)

**DOI:** 10.1007/s00431-025-06112-w

**Published:** 2025-04-17

**Authors:** Laura-Carlotta Peo, Katharina Wiehler, Johannes Paulick, Katrin Gerrer, Ariane Leone, Anja Viereck, Matthias Haegele, Silvia Stojanov, Cordula Warlitz, Silvia Augustin, Martin Alberer, Daniel B. R. Hattesohl, Laura Froehlich, Carmen Scheibenbogen, Leonard A. Jason, Lorenz L. Mihatsch, Rafael Pricoco, Uta Behrends

**Affiliations:** 1 MRI Chronic Fatigue Center for Young People (MCFC), Pediatrics, Children’S Hospital, TUM School of Medicine, Technical University of Munich, Munich, Germany; 2 MRI Chronic Fatigue Center for Young People (MCFC), Child and Adolescent Psychsomatics, Children’S Hospital, TUM School of Medicine, Technical University of Munich, Munich, Germany; 3 German Association for ME/CFS, Hamburg, Germany; 4 Research Center CATALPA, FernUniversität in Hagen, Hagen, Germany; 5 Institute of Medical Immunology, Charité - Universitätsmedizin Berlin, Corporate Member of Freie Universität Berlin and Humboldt Universität Zu Berlin and Berlin Institute of Health (BIH), Berlin, Germany; 6 Center for Community Research, Depaul University, Chicago, IL 60614 USA; 7 German Center for Infection Research (DZIF), Munich, Germany

**Correction to: European Journal of Pediatrics (2023) 183:1265–1276**

10.1007/s00431-023-05351-z

In the originally published article, entitled “Pediatric and adult patients with ME/CFS following COVID-19: A structured approach to diagnosis using the Munich Berlin Symptom Questionnaire (MBSQ)”, the Supplementary Scoring Sheets to the MBSQ, which are contained in the Supplementary File 1 (English version) and Supplementary File 2 (German version), contain an error. The dimension “*Sleep, Pain, and Neurocognitive Symptoms*” was incorrectly adopted for pediatric and adolescent patients from the Clinical Diagnostic Worksheet of Rowe, PC et al. (2017) [1].

**Published version:**

*Supplementary File 1 – English Version for Pediatric and Adolescent Patients, page 2 of the Supplementary Scoring Sheet to the MBSQ.*

**Clinical Diagnostic Worksheet by Rowe PC et al.** ^2^

**Sleep, Pain, and Neurocognitive Symptoms**

At least 2 of the following 3 categories must apply

□ At least 1 of the following 5 points must apply: **III.13–17** ≥ 2

□ At least 1 of the following 6 points must apply: **IV.18–21, V.22–43** ≥ 2

□ At least 1 of the following 8 points must apply: **V.23–25, V.27–28, V.30, V.33** ≥ 2; **V.29**: Yes

*Supplementary File 2 – German Version for Pediatric and Adolescent Patients, page 2 of the Supplementary Scoring Sheet to the MBSQ.*

**Clinical Diagnostic Worksheet by Rowe PC et al.** ^2^

**Schlaf, Schmerz und neurokognitive Symptome**

Mind. 2 der 3 Kategorien müssen erfüllt sein:

□ mind. 1 der nachfolgenden 5 Punkte muss erfüllt sein: **III.13–17** ≥ 2

□ mind. 1 der nachfolgenden 6 Punkte muss erfüllt sein: **IV.18–21, V.22–43** ≥ 2

□ mind. 1 der nachfolgenden 8 Punkte muss erfüllt sein: **V.23–25, V.27–28, V.30, V.33** ≥ 2; **V.29**: Ja

**Corrected version:**

*Supplementary File 1 – English Version for Pediatric and Adolescent Patients, page 2 of the Supplementary Scoring Sheet to the MBSQ.*

**Clinical Diagnostic Worksheet by Rowe PC et al.** ^2^

**Sleep, Pain, and Neurocognitive Symptoms**

At least 2 of the following 3 categories must apply.

□ At least 1 of the following 5 points must apply: **III.13–17** ≥ 2

□ At least 1 of the following 6 points must apply: **IV.18–21, VIII.57, VIII.59** ≥ 2

□ At least 1 of the following 8 points must apply: **V.23–25, V.27–28, V.30, V.33** ≥ 2; **V.29**: Yes

*Supplementary File 2 – German Version for Pediatric and Adolescent Patients, page 2 of the Supplementary Scoring Sheet to the MBSQ.*

**Clinical Diagnostic Worksheet by Rowe PC et al.** ^2^

**Schlaf, Schmerz und neurokognitive Symptome**

Mind. 2 der 3 Kategorien müssen erfüllt sein:

□ mind. 1 der nachfolgenden 5 Punkte muss erfüllt sein: **III.13–17** ≥ 2

□ mind. 1 der nachfolgenden 6 Punkte muss erfüllt sein: **IV.18–21, VIII.57, VIII.59** ≥ 2

□ mind. 1 der nachfolgenden 8 Punkte muss erfüllt sein: **V.23–25, V.27–28, V.30, V.33** ≥ 2; **V.29**: Ja

The original article has been corrected, and Supplementary File 1 and Supplementary File 2 have been replaced with the corrected versions.
