# Supplementary material for: Pediatric and adult patients with ME/CFS following COVID-19: A structured approach to diagnosis using the Munich Berlin Symptom Questionnaire (MBSQ)
Source: Eur J Pediatr. 2023 Dec 14;183(3):1265–76. doi: 10.1007/s00431-023-05351-z (PMC10951047; doi:10.1007/s00431-023-05351-z)
Supplement: Supplementary file 1 — Supplementary file1 (PDF 1.05 MB) [file 431_2023_5351_MOESM1_ESM.pdf]

Klinikum rechts der Isar Technical University of Munich

CHARITÉ UNIVERSITÄTSMEDIZIN BERLIN

Munich Berlin Symptom Questionnaire (MBSQ) – Questionnaire for Adults

Surname:	Name (physician):
Name:	Date (physician):
Date of birth:	Institution:
Today's date: Completion time: min	Date of disease onset:

This questionnaire has to be used in a ***medical interview.*** *Open* ***questions*** *and* ***comprehensive problems*** *should be clarified in a* ***medical visit.*** *The* ***physician's evaluation*** *of this questionnaire has to be based on the* ***supplementary scoring sheet for adults.*** *ME/CFS is a* ***clinical diagnosis.*** *The diagnosis cannot be established without appropriate* ***differential diagnostics.***

		During the last 6 months			
		**Is not present**	**Frequency**<br>**1** = sometimes<br>**2** = about ½ of the time<br>**3** = most of the time<br>**4** = always	**Severity**<br>**1** = mild<br>**2** = moderate<br>**3** = severe<br>**4** = very severe	**Physician's notes**
**I**	**Fatigue/ Daily Function**				
**1**	Fatigue (exhaustion, tiredness)	0	1 2 3 4	1 2 3 4	
**2**	Limitations in daily life	0	1 2 3 4	1 2 3 4	

**3**	If fatigue is present, is it of new or definite onset (not lifelong)?	Yes No	
**4**	If fatigue is present, is it the result of ongoing, excessive exertion?	Yes No	
**5**	If fatigue is present, is it alleviated by rest?	Yes No	

**II**	**Post-Exertional Symptoms**				
**6**	Loss of physical or mental stamina	0	1 2 3 4	1 2 3 4	
**7**	Increased symptoms after mild daily activities	0	1 2 3 4	1 2 3 4	

**8**	If post-exertional symptoms are present, how long does it take after mild daily activities until the increased symptoms have subsided?	O ≤ 1 h O 2-3 h O 4-10 h<br>O 11-13 h O 14-23 h O ≥ 24 h	
**9**	If post-exertional symptoms are present, which three mild daily activities lead to a worsening of the symptoms?	1.......................................<br>2.......................................<br>3.......................................	
**10**	If post-exertional symptoms are present, which three symptoms worsen after mild daily activities?	1.......................................<br>2.......................................<br>3.......................................	

		During the last 6 months			
		**Not present**	**Frequency**<br>**1** = sometimes<br>**2** = about ½ of the time<br>**3** = most of the time<br>**4** = always	**Severity**<br>**1** = mild<br>**2** = moderate<br>**3** = severe<br>**4** = very severe	**Physician's notes**
**III**	**Sleep**				
**11**	Unrefreshing sleep	0	1 2 3 4	1 2 3 4	
**12**	Unusually much sleep during the day	0	1 2 3 4	1 2 3 4	
**13**	Problems falling asleep	0	1 2 3 4	1 2 3 4	
**14**	Problems sleeping through	0	1 2 3 4	1 2 3 4	
**15**	Day / night reversal	0	1 2 3 4	1 2 3 4	

**IV**	**Pain**				
**16**	Myofascial pain (can include achy and sore muscles)	0	1 2 3 4	1 2 3 4	
**17**	Joint pain without swelling or redness	0	1 2 3 4	1 2 3 4	
**18**	Headaches	0	1 2 3 4	1 2 3 4	

**V**	**Neurocognitive Manifestations**				
**19**	Confusion	0	1 2 3 4	1 2 3 4	
**20**	Slowness of thought	0	1 2 3 4	1 2 3 4	
**21**	Concentration problems	0	1 2 3 4	1 2 3 4	
**22**	Short-term memory problems	0	1 2 3 4	1 2 3 4	

		During the last 6 months			
		Not present	Frequency<br>**1** = sometimes<br>**2** = about ½ of the time<br>**3** = most of the time<br>**4** = always	Severity<br>**1** = mild<br>**2** = moderate<br>**3** = severe<br>**4** = very severe	**Physician's notes**
**23**	Orientation problems	0	1 2 3 4	1 2 3 4	
**24**	Comprehension problems / attention problems	0	1 2 3 4	1 2 3 4	
**25**	Word-finding problems	0	1 2 3 4	1 2 3 4	
**26**	If cognitive symptoms (**V.20-25**) are present, do they worsen due to effort, upright body position, stress or time pressure?		Yes No		
**27**	Perceptual and sensory disturbances, e.g., blurred vision	0	1 2 3 4	1 2 3 4	
**28**	Coordination problems	0	1 2 3 4	1 2 3 4	
**29**	Muscle twitches	0	1 2 3 4	1 2 3 4	
**30**	Muscle weakness	0	1 2 3 4	1 2 3 4	
**31**	Overload phenomena - hypersensitivity to (bright) light	0	1 2 3 4	1 2 3 4	
**32**	Overload phenomena - hypersensitivity to noise	0	1 2 3 4	1 2 3 4	
**33**	Overload phenomena - hypersensitivity to touch	0	1 2 3 4	1 2 3 4	
**34**	Overload phenomena – emotional overload	0	1 2 3 4	1 2 3 4	
**VI**	**Autonomic Manifestations**				
**35**	Dizziness	0	1 2 3 4	1 2 3 4	
**36**	Palpitations with or without cardiac arrhythmias	0	1 2 3 4	1 2 3 4	
**37**	Circulatory problems when getting up	0	1 2 3 4	1 2 3 4	
**38**	Circulatory problems in an upright posture	0	1 2 3 4	1 2 3 4	
**39**	Unusual paleness	0	1 2 3 4	1 2 3 4	
**40**	Bladder problems	0	1 2 3 4	1 2 3 4	
**41**	Gastrointestinal problems	0	1 2 3 4	1 2 3 4	
**42**	Exertional shortness of breath	0	1 2 3 4	1 2 3 4	
**VII**	**Neuroendocrine Manifestations**				
**43**	Loss of thermostatic stability (e.g., subnormal body temperature, and marked diurnal fluctuation, sweating episodes, feelings of feverishness, cold limbs)	0	1 2 3 4	1 2 3 4	
**44**	Intolerance of extremes hot and cold	0	1 2 3 4	1 2 3 4	
**45**	Marked weight change with loss of appetite or abnormal appetite	0	1 2 3 4	1 2 3 4	
**46**	Worsening of symptoms with stress	0	1 2 3 4	1 2 3 4	
**VIII**	**Immunologic Manifestations**				
**47**	Flu-like symptoms / general malaise	0	1 2 3 4	1 2 3 4	
**48**	Painful / sensitive lymph nodes	0	1 2 3 4	1 2 3 4	
**49**	Recurrent sore throat	0	1 2 3 4	1 2 3 4	
**50**	New sensitivities to food, medication or chemicals etc.	0	1 2 3 4	1 2 3 4	

**Further Questions About the Symptom Course**		
If there are any symptoms,<br>what are the three main symptoms of this questionnaire?	1……………………………………………………………………………<br>2……………………………………………………………………………<br>3……………………………………………………………………………	

MBSQ_questionnaire_adults_English_2021 07 29_K. Wiehler, J. Paulick, R. Pricoco, A. Leone, D.B.R. Hattesohl, L. Fröhlich, C. Scheibenbogen, U. Behrends ©

Klinikum rechts der Isar Technical University of Munich

CHARITÉ UNIVERSITÄTSMEDIZIN BERLIN

Munich Berlin Symptom Questionnaire (MBSQ) – Scoring Sheet for Adults (≥ 18 Years of Age)

Surname:
Name:
Date of birth:
Today's date:

Surname (physician):
Name (physician):
Date (physician):
Institution:

Only if frequency **and** severity are reported with **≥2** after the physician's evaluation, the respective item counts positively for evaluation. ME/CFS is a diagnosis of exclusion that needs to be reevaluated in case of new clinical aspects.

	**Canadian Consensus Criteria**[1]		**IOM Criteria**[2]
O	**Duration of Illness (Onset of the Symptoms: ____.____.________)**<br>The symptomatology is present for at least **6 months**.	O	**Duration of Illness (Onset of the Symptoms: ____.____.________)**<br>The symptomatology is present for at least **6 months.**
O	**Medical History, Physical Examination, and Differential Diagnostics**<br>☐ Did not indicate any other cause for the symptoms (in particular, no indications for *Addison's disease, Cushing's disease, hypo/ hyperthyroidism, anemia, hemochromatosis, diabetes mellitus, haemato-oncological, rheumatological and treatable sleep disorders)*<br>☐ The symptoms must have begun or have been significantly altered after the onset of the illness.	O	**Medical History, Physical Examination, and Differential Diagnostics**<br>☐ Did not indicate any other cause for the symptoms<br>☐ The symptoms must have begun or have been significantly altered after the onset of the illness.
O	**Fatigue / Daily Function**<br>All of the following points must apply:<br>☐ Fatigue: **I.1** ≥2<br>☐ Limitations in daily life: **I.2** ≥2<br>☐ Fatigue new start: **I.3** Yes	O	**Fatigue / Daily Function**<br>All of the following points must apply:<br>☐ Fatigue: **I.1** ≥2<br>☐ Limitations in daily life: **I.2** ≥2<br>☐ Fatigue new start: **I.3** Yes<br>☐ Fatigue due to exertion: **I.4** No<br>☐ Fatigue alleviated by rest: **I.5** No
O	**Post-Exertional Symptoms**<br>All of the following points must apply:<br>☐ Loss of stamina; increased symptoms after everyday activities: all 2 points **II.6-7** ≥2<br>☐ Duration of worsening of symptoms: **II.8** ≥14 hours*	O	**Post-Exertional Symptoms**<br>All of the following points must apply: all 2 points **II.6-7** ≥2
O	**Sleep**<br>At least 1 of the following 5 points must apply: **III.11-15** ≥2	O	**Sleep**<br>The following point must apply: **III.11** ≥2

	Canadian Consensus Criteria[1]		IOM Criteria [2]
O	**Pain**<br>At least 1 of the following 3 points must apply: **IV.16-18** ≥2		**Pain**<br>*Not included in IOM criteria*
O	**Neurocognitive Manifestations**<br>At least 2 of the following symptom groups must apply:<br>☐ Confusion: **V.19** ≥2<br>☐ Concentration and/or Memory: 1 of the following 2 points: **V.21-22** ≥2<br>☐ Orientation: **V.23** ≥2<br>☐ Comprehension and/or word-finding: 1 of the following 2 points: **V.24-25** ≥2<br>☐ Perception and overload phenomena: 1 of the following 5 points: **V.27, V.31-34** ≥2<br>☐ Coordination and muscular system: 1 of the following 3 points: **V.28-30** ≥2	O	**Neurocognitive and Autonomous Manifestations**<br>At least 1 of the following 2 categories must apply:<br>☐ The 2 following symptom groups must apply:<br>☐ At least 1 of the following 6 points must apply: **V.20-22, V.24-25, V.28** ≥2<br>☐ The following point must apply: **V.26** Yes<br>☐ At least 1 of the following 2 points must apply: **VI.37-38** ≥2
O	**Autonomic, Neuroendocrine, Immunological Manifestations**<br>At least 2 of the following 3 categories must apply:<br>☐ At least 1 of the following 8 points must apply: **VI.35-42** ≥2<br>☐ At least 1 of the following 4 points must apply: **VII.43-46** ≥2<br>☐ At least 1 of the following 4 points must apply: **VIII.47-50**, ≥2		**Neuroendocrine, Immunological Manifestations**<br>*Not included in IOM criteria*

O **Patient meets the Canadian Consensus Criteria for ME/CFS**	O **Patient meets the IOM criteria for ME/CFS**
O **Patient does <u>not</u> meet the Canadian Consensus Criteria for ME/CFS**	O **Patient does <u>not</u> meet the IOM criteria for ME/CFS**

[1] Carruthers BM, Jain AK, De Meirleir KL, Peterson DL, Klimas NG, Lerner, AM, Bested AC, Flor-Henry P, Joshi P, Powles ACP, Sherkey JA, van de Sande MI. Myalgic Encephalomyelitis/Chronic Fatigue Syndrome. Journal of Chronic Fatigue Syndrome 2003;11:7-115. doi: 10.1300/J092v11n01_02.

[2] Clayton EW. Beyond Myalgic Encephalomyelitis/Chronic Fatigue Syndrome: An IOM Report on Redefining an Illness. JAMA. 2015;313(11):1101–1102. doi:10.1001/jama.2015.1346

* In the original publication of Carruthers BM et al. PEM requires a duration of >24 hours. However, according to the current state of international research, we recommend diagnosing PEM with a duration of ≥14 hours, see
a) Cotler J, Holtzman C, Dudun C, Jason LA. A Brief Questionnaire to Assess Post-Exertional Malaise. Diagnostics. 2018; 8(3):66. doi.org/10.3390/diagnostics8030066
b) Kedor C, Freitag H, Meyer-Arndt L, Wittke K, Hanitsch LG, Zoller T, Steinbeis F, Haffke M, Rudolf G, Heidecker B, Bobbert T, Spranger J, Volk HD, Skurk C, Konietschke F, Paul F, Behrends U, Bellmann-Strobl J, Scheibenbogen C. A prospective observational study of post-COVID-19 chronic fatigue syndrome following the first pandemic wave in Germany and biomarkers associated with symptom severity. Nat Commun. 2022;13(1):5104. doi: 10.1038/s41467-022-32507-6. Erratum in: Nat Commun. 2022;13(1):6009.

Klinikum rechts der Isar Technical University of Munich

CHARITÉ UNIVERSITÄTSMEDIZIN BERLIN

Munich Berlin Symptom Questionnaire (MBSQ) – Questionnaire for Pediatric and Adolescent Patients

Surname:	Name (physician):
Name:	Date (physician):
Date of birth:	Institution:
Today's date: Completion time: min	Date of disease onset:

<u>For patients</u>: Please complete this questionnaire on your own as far as possible. If necessary, ask your parents to help you.
*<u>For doctors</u>: This questionnaire has to be used in a **medical interview.** Open **questions** and comprehensive **problems** should be clarified in a **medical visit.** The **physician's evaluation** of this questionnaire has to be based on the **supplementary scoring sheet for pediatric and adolescent patients.** ME/CFS is a **clinical diagnosis.** The diagnosis cannot be established without appropriate **differential diagnostics.***

		During the last 3 months			
		**Is not present**	**Frequency**<br>**1** = sometimes<br>**2** = about ½ of the time<br>**3** = most of the time<br>**4** = always	**Severity**<br>**1** = mild<br>**2** = moderate<br>**3** = severe<br>**4** = very severe	**Physician's notes**
**I**	**Fatigue / Daily Function**				
**1**	Fatigue (exhaustion, tiredness)	0	1 2 3 4	1 2 3 4	
**2**	Limitations in daily life - school/ education	0	1 2 3 4	1 2 3 4	
**3**	Limitations in daily life - social	0	1 2 3 4	1 2 3 4	
**4**	Limitations in daily life - personal	0	1 2 3 4	1 2 3 4	

**5**	<u>If</u> fatigue is present, is it of new or definite onset (not lifelong)?	Yes No	
**6**	<u>If</u> fatigue is present, is it the result of ongoing, excessive exertion?	Yes No	
**7**	<u>If</u> fatigue is present, is it alleviated by rest?	Yes No	

**II**	**Post-Exertional Symptoms**				
**8**	Loss of physical or mental stamina	0	1 2 3 4	1 2 3 4	
**9**	Increased symptoms after light everyday activities	0	1 2 3 4	1 2 3 4	

**10**	<u>If</u> post-exertional symptoms are present, how long does it take after mild daily activities until the increased symptoms have subsided?	O ≤ 1 h O 2-3 h O 4-10 h<br>O 11-13 h O 14-23 h O ≥ 24 h	
**11**	<u>If</u> post-exertional symptoms are present, which three mild daily activities lead to a worsening of the symptoms?	1.................................<br>2.................................<br>3.................................	
**12**	<u>If</u> post-exertional symptoms are present, which three symptoms worsen after mild daily activities?	1.................................<br>2.................................<br>3.................................	

		During the last 3 months			
		**Not present**	**Frequency**<br>**1** = sometimes<br>**2** = about ½ of the time<br>**3** = most of the time<br>**4** = always	**Severity**<br>**1** = mild<br>**2** = moderate<br>**3** = severe<br>**4** = very severe	**Physician's notes**
**III**	**Sleep**				
**13**	Unrefreshing sleep	0	1 2 3 4	1 2 3 4	
**14**	Unusually much sleep during the day	0	1 2 3 4	1 2 3 4	
**15**	Problems falling asleep	0	1 2 3 4	1 2 3 4	
**16**	Problems sleeping through	0	1 2 3 4	1 2 3 4	
**17**	Day/ night reversal	0	1 2 3 4	1 2 3 4	

**IV**	**Pain**				
**18**	Myofascial pain (can include achy and sore muscles)	0	1 2 3 4	1 2 3 4	
**19**	Joint pain without swelling or redness	0	1 2 3 4	1 2 3 4	
**20**	Headaches	0	1 2 3 4	1 2 3 4	
**21**	Abdominal pain	0	1 2 3 4	1 2 3 4	

	Not present	Frequency	Severity	Physician's notes
	**During the last 3 months**			
		**Frequency**<br>**1** = sometimes<br>**2** = about ½ of the time<br>**3** = most of the time<br>**4** = always	**Severity**<br>**1** = mild<br>**2** = moderate<br>**3** = severe<br>**4** = very severe	
**V Neurocognitive Manifestations**				
**22** Confusion	0	1 2 3 4	1 2 3 4	
**23** Slowness of thought	0	1 2 3 4	1 2 3 4	
**24** Concentration problems	0	1 2 3 4	1 2 3 4	
**25** Short-term memory problems	0	1 2 3 4	1 2 3 4	
**26** Orientation problems	0	1 2 3 4	1 2 3 4	
**27** Comprehension problems / attention problems	0	1 2 3 4	1 2 3 4	
**28** Word-finding problems	0	1 2 3 4	1 2 3 4	

**29** If cognitive symptoms (**V.23-28**) are present, do they worsen due to effort, upright body position, stress or time pressure?	Yes No	

**30** Absent mindedness	0	1 2 3 4	1 2 3 4	
**31** Difficulty recalling information	0	1 2 3 4	1 2 3 4	
**32** Need to focus on one thing at a time	0	1 2 3 4	1 2 3 4	
**33** Trouble expressing thought	0	1 2 3 4	1 2 3 4	
**34** Lose train of thought	0	1 2 3 4	1 2 3 4	
**35** New troubles with math or other educational subjects	0	1 2 3 4	1 2 3 4	
**36** Perceptual and sensory disturbances, e.g., blurred vision	0	1 2 3 4	1 2 3 4	
**37** Coordination problems	0	1 2 3 4	1 2 3 4	
**38** Muscle twitches	0	1 2 3 4	1 2 3 4	
**39** Muscle weakness	0	1 2 3 4	1 2 3 4	
**40** Overload phenomena - hypersensitivity to (bright) light	0	1 2 3 4	1 2 3 4	
**41** Overload phenomena - hypersensitivity to noise	0	1 2 3 4	1 2 3 4	
**42** Overload phenomena - hypersensitivity to touch	0	1 2 3 4	1 2 3 4	
**43** Overload phenomena – emotional overload	0	1 2 3 4	1 2 3 4	

**VI Autonomic Manifestations**				
**44** Dizziness	0	1 2 3 4	1 2 3 4	
**45** Palpitations with or without cardiac arrhythmias	0	1 2 3 4	1 2 3 4	
**46** Circulatory problems when getting up	0	1 2 3 4	1 2 3 4	
**47** Circulatory problems in upright posture	0	1 2 3 4	1 2 3 4	
**48** Unusual paleness	0	1 2 3 4	1 2 3 4	
**49** Bladder problems	0	1 2 3 4	1 2 3 4	
**50** Gastrointestinal problems	0	1 2 3 4	1 2 3 4	
**51** Exertional shortness of breath	0	1 2 3 4	1 2 3 4	

**VII Neuroendocrine Manifestations**				
**52** Loss of thermostatic stability (e.g., subnormal body temperature, and marked diurnal fluctuation, sweating episodes, feelings of feverishness, cold limbs)	0	1 2 3 4	1 2 3 4	
**53** Intolerance of extremes hot and cold	0	1 2 3 4	1 2 3 4	
**54** Marked weight change with loss of appetite or abnormal appetite	0	1 2 3 4	1 2 3 4	
**55** Worsening of symptoms with stress	0	1 2 3 4	1 2 3 4	

**VIII Immunologic Manifestations**				
**56** Flu-like symptoms / general malaise	0	1 2 3 4	1 2 3 4	
**57** Painful / sensitive lymph nodes	0	1 2 3 4	1 2 3 4	
**58** Fever	0	1 2 3 4	1 2 3 4	
**59** Recurrent sore throat	0	1 2 3 4	1 2 3 4	
**60** New sensitivities to food, medication or chemicals etc.	0	1 2 3 4	1 2 3 4	

**Further Questions About the Symptom Course**		
If there are any symptoms,<br>what are the three main symptoms of this questionnaire?	1..<br>2..<br>3..	

MBSQ_questionnaire_children_English_2021 08 09_K. Wiehler, J. Paulick, R. Pricoco, A. Leone, D.R.B. Hattesohl, L. Fröhlich, C. Scheibenbogen, U. Behrends ©

Klinikum rechts der Isar Technical University of Munich

CHARITÉ UNIVERSITÄTSMEDIZIN BERLIN

Munich Berlin Symptom Questionnaire (MBSQ) – Scoring Sheet No. 1 for Children and Adolescents (0-17 Years)

Surname:	Surname (physician):
Name:	Name (physician):
Date of birth:	Date (physician):
Today's date:	Institution:

Only if frequency **and** severity are reported with ≥**2** after the physician's evaluation, the respective item counts positively for evaluation. ME/CFS is a diagnosis of exclusion that needs to be reevaluated in case of new clinical aspects.

	Pediatric Case Definition for ME/CFS by Jason LA et al.[1]		Clinical Diagnostic Worksheet by Rowe PC et al.[2]
O	**Duration of Illness (Onset of the Symptoms: ____.____.________)**<br>The symptomatology is present for at least **3 months**.	O	**Duration of Illness (Onset of the Symptoms: ____.____.________)**<br>The symptomatology is present for at least **3 months**.*
O	**Medical History, Physical Examination and Differential Diagnostics**<br>Did not indicate any other cause for the symptoms<br>(in particular, no indications for *untreated hypothyroidism, sleep apnea, narcolepsy, malignancies, leukemia, unresolved hepatitis, multiple sclerosis, juvenile rheumatoid arthritis, lupus erythematosus, HIV/ AIDS, severe obesity (BMI greater than 40), celiac disease, lyme disease, childhood schizophrenia or psychiatric disorders, bipolar disorder, active alcohol or substance abuse, active anorexia nervosa or bulimia nervosa, depressive disorders)*	O	**Medical History, Physical Examination and Differential Diagnostics**<br>Did not indicate any other cause for the symptoms<br>(in particular, no evidence of *adrenocortical insufficiency, overtraining syndrome, GI disorders: Celiac disease, irritable bowel syndrome, eosinophilic gastroenteritis; Chiari malformation, cervical spinal stenosis, neuroborreliosis or other tick-borne disease, major depression, narcolepsy, obstructive or central sleep apnea, postcommotion syndrome, severe anemia, systemic lupus erythematosus and similar autoimmune disease, untreated hypo-/hyperthyroidism).*
O	**Fatigue / Daily Function**<br>All of the following points must apply:<br>☐ Fatigue: **I.1** ≥2<br>☐ Limitations: all 3 points: **I.2-4** ≥2<br>☐ Fatigue due to exertion: **I.6** No<br>☐ Fatigue alleviated by rest: **I.7** No	O	**Fatigue / Daily Function**<br>All of the following points must apply:<br>☐ Fatigue: **I.1** ≥2<br>☐ Limitations: at least 1 of the 3 points: **I.2-4** ≥2<br>☐ Fatigue due to exertion: **I.6** No<br>☐ Fatigue alleviated by rest: **I.7** No
O	**Post-Exertional Symptoms**<br>All of the following points must apply:<br>☐ loss of stamina; increased symptoms after everyday activities: all 2 points: **II.8-9** ≥2<br>☐ duration of worsening of symptoms: **II.10** ≥14 hours	O	**Post-Exertional Symptoms**<br>All of the following points must apply:<br>☐ loss of stamina; increased symptoms after everyday activities: all 2 points: **II.8-9** ≥2<br>☐ duration of worsening of symptoms: **II.10** >24 hours

Pediatric Case Definition for ME/CFS by Jason LA et al.[1]		Clinical Diagnostic Worksheet by Rowe PC et al.[2]	
O	**Sleep**<br>At least 1 of the following 5 points must apply: **III.13-17** ≥2	O	**Sleep, Pain, and Neurocognitive Symptoms**<br>At least 2 of the following 3 categories must apply:<br>☐ At least 1 of the following 5 points must apply: **III.13-17** ≥2<br>☐ At least 1 of the following 4 points must apply: **IV.18-21, VIII.57, VIII.59** ≥2<br>☐ At least 1 of the following 8 points must apply: **V.23-25, V.27-28, V.30, V.33** ≥2; **V.29**: Yes
O	**Pain**<br>At least 1 of the following 4 points must apply: **IV.18-21** ≥2		
O	**Neurocognitive Manifestations**<br>At least 2 of the following 11 points must apply: **V.23-25; V.27-28; V.30-35** ≥2		
O	**Autonomic, Neuroendocrine and Immunologic Manifestations**<br>At least 2 of the following 3 categories must apply:<br>☐ At least 1 of the following 5 points must apply: **VI.44-47; VI.51** ≥2<br>☐ At least 1 of the following 4 points must apply: **VII.52-55** ≥2<br>☐ At least 1 of the following 4 points must apply: **VIII.56-59** ≥2	O	**Autonomic, Neuroendocrine, and Immunologic Manifestations**<br>*Not included in the Clinical Diagnostic Worksheet by Rowe PC et al.*

O **Patient meets the Pediatric Case Definition for ME/CFS by Jason LA et al.**	O **Patient meets the Clinical Diagnostic Worksheet criteria for ME/CFS by Rowe PC et al.**
O **Patient does <u>not</u> meet the Pediatric Case Definition for ME/CFS by Jason LA et al.**	O **Patient does <u>not</u> meet the Clinical Diagnostic Worksheet criteria for ME/CFS by Rowe PC et al.**

[1]Jason LA, Jordan K, Miike T, Bell DS, Lapp C, Torres-Harding S, Rowe K, Gurwitt A, De Meirleir K, Van Hoof ELS. Pediatric Case Definition for Myalgic Encephalomyelitis and Chronic Fatigue Syndrome. Journal of Chronic Fatigue Syndrome 2006; 13:1-44. doi: 10.1300/J092v13n02_01.

[2] Rowe PC, Underhill RA, Friedman KJ, Gurwitt A, Medow MS, Schwartz MS, Speight N, Stewart JM, Vallings R, Rowe KS. Myalgic Encephalomyelitis/Chronic Fatigue Syndrome Diagnosis and Management in Young People: A Primer. Front Pediatr. 2017;5:121. doi: 10.3389/fped.2017.00121.

* In the original publication by Rowe PC et al. a duration of at least 6 months is required for the diagnosis. If the duration of the disease is <6 months, it is only recommended to express an urgent suspicion. However, we recommend a diagnosis in children and adolescents with a disease duration of only 3 months in order to provide early symptom-oriented care.

Klinikum rechts der Isar | Technical University of Munich

CHARITÉ UNIVERSITÄTSMEDIZIN BERLIN

Munich Berlin Symptom Questionnaire (MBSQ) – Scoring Sheet No. 2 for Children and Adolescents (0-17 Years)

Surname:	Surname (physician):
Name:	Name (physician):
Date of birth:	Date (physician):
Today's date:	Institution:

Only if frequency **and** severity are reported with ≥**2** after the physician's evaluation, the respective item counts positively for evaluation. ME/CFS is a diagnosis of exclusion that needs to be reevaluated in case of new clinical aspects.

Canadian Consensus Criteria[3]	IOM Criteria[4]
O **Duration of Illness (Onset of the Symptoms: ____.____.________)**<br>The symptomatology has been present for at least **3 months**.	O **Duration of Illness (Onset of the Symptoms: ____.____.________)**<br>The symptomatology has been present for at least **3 months**.**
O **Medical History, Physical Examination and Differential Diagnostics**<br>☐ Did not indicate any other cause for the symptoms *(in particular no indications for Addison's disease, Cushing's disease, hypo-/hyperthyroidism, anemia, hemochromatosis, diabetes mellitus, haemato-oncological, rheumatological and treatable sleep disorders)*<br>☐ The symptoms must have begun or have been significantly altered after the onset of the illness.	O **Medical History, Physical Examination and Differential Diagnostics**<br>☐ Did not indicate any other cause for the symptoms<br>☐ The symptoms must have begun or have been significantly altered after the onset of the illness.
O **Fatigue / Daily Function**<br>All of the following points must apply:<br>☐ Fatigue: **I.1** ≥2<br>☐ Limitations: at least 1 of the 3 points: **I.2-4** ≥2<br>☐ Fatigue of definite start: **I.5** Yes	O **Fatigue / Daily Function**<br>All of the following points must apply:<br>☐ Fatigue: **I.1** ≥2<br>☐ Limitations: at least 1 of the 3 points: **I.2-4** ≥2<br>☐ Fatigue new start: **I.5** Yes<br>☐ Fatigue due to exertion: **I.6** No<br>☐ Fatigue alleviated by rest: **I.7** No
O **Post-Exertional Symptoms**<br>All of the following points must apply:<br>☐ Loss of stamina; increased symptoms after everyday activities: all 2 points **II.8-9** ≥2<br>☐ Duration of worsening of symptoms: **II.10** ≥14 hours*	O **Post-Exertional Symptoms**<br>All of the following points must apply: all 2 points: **II.8-9** ≥2

Canadian Consensus Criteria[3]	IOM Criteria[4]
O **Sleep**<br>At least 1 of the following 5 points must apply: **III.13-17** ≥2	O **Sleep**<br>The following point must apply: **III.13** ≥2
O **Pain**<br>At least 1 of the following 3 points must apply: **IV.18-20** ≥2 *(except abdominal pain)*	O **Pain**<br>*Not included in IOM criteria*
O **Neurocognitive Manifestations**<br>At least 2 of the following symptom groups must apply:<br>☐ Confusion: **V.22** ≥2<br>☐ Concentration and/or Memory: 1 of the following 2 points: **V.24-25** ≥2<br>☐ Orientation: **V.26** ≥2<br>☐ Comprehension and/or word-finding: 1 of the following 2 points: **V.27-28** ≥2<br>☐ Perception and overload phenomena: 1 of the following 5 points: **V.36**, **V.40-43** ≥2<br>☐ Coordination und muscular system: 1 of the following 3 points: **V.37-39** ≥2	O **Neurocognitive and Autonomic Manifestations**<br>At least 1 of the following 2 categories must apply:<br>☐ The 2 following symptom groups must apply:<br>☐ At least 1 of the following 6 points must apply: **V.23-25, V.27-28**, **V.37** ≥2<br>☐ The following point must apply: **V.29**, Yes<br>☐ At least 1 of the following 2 points must apply: **VI.46-47** ≥2
O **Autonomic, Neuroendocrine, Immunologic Manifestations**<br>At least 2 of the following 3 categories must apply:<br>☐ At least 1 of the following 8 points must apply: **VI.44-51** ≥2<br>☐ At least 1 of the following 4 points must apply: **VII.52-55** ≥2<br>☐ At least 1 of the following 4 points must apply: **VIII.56-57, VIII.59-60** ≥2	O **Neuroendocrine, Immunologic Manifestations**<br>*Not included in IOM criteria*

O **Patient meets the Canadian Consensus Criteria for ME/CFS**<br><br>O **Patient does <u>not</u> meet the Canadian Consensus Criteria for ME/CFS**	O **Patient meets the IOM criteria for ME/CFS**<br><br>O **Patient does <u>not</u> meet the IOM criteria for ME/CFS**

[3] Carruthers BM, Jain AK, De Meirleir KL, Peterson DL, Klimas NG, Lerner, AM, Bested AC, Flor-Henry P, Joshi P, Powles ACP, Sherkey JA, van de Sande MI. Myalgic Encephalomyelitis/Chronic Fatigue Syndrome. Journal of Chronic Fatigue Syndrome 2003;11:7-115. doi: 10.1300/J092v11n01_02.

[4] Clayton EW. Beyond Myalgic Encephalomyelitis/Chronic Fatigue Syndrome: An IOM Report on Redefining an Illness. JAMA. 2015;313(11):1101–1102. doi:10.1001/jama.2015.1346.

* In the original publication of Carruthers BM et al. PEM requires a duration of >24 hours. However, according to the current state of international research, we recommend diagnosing PEM with a duration of ≥14 hours, see a) Cotler J et al. Diagnostics. 2018;8(3):66. doi.org/10.3390/diagnostics8030066 and b) Kedor C et al., Nat Commun. 2022;13(1):5104. doi: 10.1038/s41467-022-32507-6. Erratum in: Nat Commun. 2022;13(1):6009.

** In the original publication by Clayton EW et al.[4] a duration of at least 6 months is required for the diagnosis. However, we recommend diagnosing children and adolescents after 3 months to provide them with symptom-oriented care at an early stage.
