# Supplementary material for: Pediatric and adult patients with ME/CFS following COVID-19: A structured approach to diagnosis using the Munich Berlin Symptom Questionnaire (MBSQ)
Source: Eur J Pediatr. 2023 Dec 14;183(3):1265–76. doi: 10.1007/s00431-023-05351-z (PMC10951047; doi:10.1007/s00431-023-05351-z)
Supplement: Supplementary file 2 — Supplementary file2 (PDF 1.10 MB) [file 431_2023_5351_MOESM2_ESM.pdf]

Klinikum rechts der Isar   Technische Universität München

CHARITÉ UNIVERSITÄTSMEDIZIN BERLIN

Munich Berlin Symptom Questionnaire (MBSQ) – Ärztlicher Anamnesebogen für Erwachsene bei Verdacht auf Myalgische Enzephalomyelitis / Chronisches Fatigue-Syndrom (ME/CFS)

Name:	Vorname:		Name (Arzt*in):
Geburtsdatum:			Datum (Arzt*in):
Ausfülldatum:	Ausfülldauer:	min	Einrichtung:

*Dieser Bogen stellt die Grundlage für **ein ärztliches Gespräch** dar. **Offene Punkte** oder **Verständnisprobleme** müssen im **ärztlichen Gespräch** geklärt werden. Die **ärztliche Beurteilung** erfolgt anhand des **Auswertungsbogens für Erwachsene**. ME/CFS ist eine **klinische Diagnose**. Die Diagnosestellung erfordert zusätzlich zu dieser Erhebung eine **angemessene Differenzialdiagnostik**.*

		**Während der letzten 6 Monate**			
		**Liegt nicht vor**	**Häufigkeit**<br>**1** = manchmal<br>**2** = etwa ½ der Zeit<br>**3** = meistens<br>**4** = immer	**Schwere**<br>**1** = mild<br>**2** = moderat<br>**3** = schwer<br>**4** = sehr schwer	**Ärztlicher Vermerk**
**I**	**Fatigue**				
**1**	Fatigue (Erschöpfung, Abgeschlagenheit, Schlappheit, Schwäche, Mangel an Energie)	0	1 2 3 4	1 2 3 4	
**2**	(Leistungs-)Einschränkungen im Alltag	0	1 2 3 4	1 2 3 4	

Falls Fatigue vorliegt:			
**3**	Die Fatigue hat zu einem neuen oder bestimmten Zeitpunkt begonnen (nicht lebenslang).	O Trifft zu. O Trifft nicht zu.	
**4**	Die Fatigue ist **nicht** Folge von anhaltender, exzessiver Belastung.	O Trifft zu. O Trifft nicht zu.	
**5**	Die Fatigue bessert sich erheblich durch Ausruhen.	O Trifft zu. O Trifft nicht zu.	

**II**	**Belastungsintoleranz / belastungsinduzierte Symptomverschlechterung**				
**6**	Vermindertes geistiges oder körperliches Durchhaltevermögen	0	1 2 3 4	1 2 3 4	
**7**	Vermehrte Beschwerden nach alltäglichen Aktivitäten (körperlicher, geistiger oder emotionaler Art)	0	1 2 3 4	1 2 3 4	

Falls Belastungsintoleranz vorliegt:					
**8**	Wie lange dauert es nach alltäglichen Aktivitäten bis die vermehrten Beschwerden wieder abgeklungen sind?	O ≤ 1 Std.<br>O 11-13 Std.	O 2-3 Std.<br>O 14-23 Std.	O 4-10 Std.<br>O ≥ 24 Std.	
**9**	Welche drei alltäglichen Aktivitäten führen zu vermehrten Beschwerden?	1.......................................<br>2.......................................<br>3.......................................			
**10**	Welche drei Symptome verschlechtern sich nach alltäglichen Aktivitäten?	1.......................................<br>2.......................................<br>3.......................................			

**III**	**Schlaf**				
**11**	Unerholsamer Schlaf	0	1 2 3 4	1 2 3 4	
**12**	Ungewöhnlich viel Schlaf tagsüber	0	1 2 3 4	1 2 3 4	
**13**	Einschlafprobleme	0	1 2 3 4	1 2 3 4	
**14**	Durchschlafprobleme	0	1 2 3 4	1 2 3 4	
**15**	Verschobener Tag-Nacht-Rhythmus	0	1 2 3 4	1 2 3 4	

**IV**	**Schmerzen**				
**16**	Muskelschmerzen, muskelkaterartiges Gefühl	0	1 2 3 4	1 2 3 4	
**17**	Gelenkschmerzen ohne Schwellung oder Rötung	0	1 2 3 4	1 2 3 4	
**18**	Kopfschmerzen	0	1 2 3 4	1 2 3 4	

	Während der letzten 6 Monate			
	Liegt nicht vor	Häufigkeit<br>**1** = manchmal<br>**2** = etwa ½ der Zeit<br>**3** = meistens<br>**4** = immer	Schwere<br>**1** = mild<br>**2** = moderat<br>**3** = schwer<br>**4** = sehr schwer	Ärztlicher Vermerk
**V Neurokognitive Manifestationen**				
**19** Verwirrung	0	1 2 3 4	1 2 3 4	
**20** Verlangsamtes Denken	0	1 2 3 4	1 2 3 4	
**21** Konzentrationsprobleme	0	1 2 3 4	1 2 3 4	
**22** Probleme mit dem Kurzzeitgedächtnis	0	1 2 3 4	1 2 3 4	
**23** Orientierungsprobleme	0	1 2 3 4	1 2 3 4	
**24** Verständnisprobleme / Aufmerksamkeitsprobleme	0	1 2 3 4	1 2 3 4	
**25** Wortfindungsstörungen	0	1 2 3 4	1 2 3 4	

**26** Falls kognitive Beschwerden (**V.20-25**) vorliegen, verschlechtern sich diese durch Anstrengung, anhaltende aufrechte Position, Stress oder Zeitdruck?	Ja Nein	

**27** Wahrnehmungs-, Empfindungs- und/oder Sinnesstörungen (z.B. verschwommenes Sehen)	0	1 2 3 4	1 2 3 4	
**28** Koordinationsschwierigkeiten	0	1 2 3 4	1 2 3 4	
**29** Muskelzuckungen	0	1 2 3 4	1 2 3 4	
**30** Muskelschwäche	0	1 2 3 4	1 2 3 4	
**31** Lichtempfindlichkeit	0	1 2 3 4	1 2 3 4	
**32** Geräuschempfindlichkeit	0	1 2 3 4	1 2 3 4	
**33** Berührungsempfindlichkeit	0	1 2 3 4	1 2 3 4	
**34** Überlastung durch positive und / oder negative Emotionen	0	1 2 3 4	1 2 3 4	

**VI Autonome Manifestationen**				
**35** Schwindel / Benommenheit	0	1 2 3 4	1 2 3 4	
**36** Herzklopfen / Herzrasen	0	1 2 3 4	1 2 3 4	
**37** Kreislaufprobleme beim Aufrichten	0	1 2 3 4	1 2 3 4	
**38** Kreislaufprobleme in aufrechter Position	0	1 2 3 4	1 2 3 4	
**39** Ungewohnte Blässe	0	1 2 3 4	1 2 3 4	
**40** Blasenprobleme	0	1 2 3 4	1 2 3 4	
**41** Magen-Darm-Probleme	0	1 2 3 4	1 2 3 4	
**42** Belastungsabhängige Atembeschwerden	0	1 2 3 4	1 2 3 4	

**VII Neuroendokrine Manifestationen**				
**43** Temperaturregulationsstörung (z.B. erniedrigte Temperatur, Temperaturschwankungen, Schwitzen, fiebriges Gefühl, kalte Hände / Füße)	0	1 2 3 4	1 2 3 4	
**44** Unverträglichkeit von extremer Hitze / Kälte	0	1 2 3 4	1 2 3 4	
**45** Gewichtsveränderung bei Appetitveränderung	0	1 2 3 4	1 2 3 4	
**46** Symptomverschlechterung bei Stress	0	1 2 3 4	1 2 3 4	

**VIII Immunologische Manifestationen**				
**47** Grippiges Gefühl / generelles Krankheitsgefühl	0	1 2 3 4	1 2 3 4	
**48** Schmerzhafte / empfindliche Lymphknoten	0	1 2 3 4	1 2 3 4	
**49** Wiederkehrende Halsschmerzen	0	1 2 3 4	1 2 3 4	
**50** Neue Empfindlichkeit gegenüber Lebensmitteln, Medikamenten, Chemikalien, etc.	0	1 2 3 4	1 2 3 4	

**Hauptsymptome**		
Falls bei Ihnen mehr als drei der genannten Symptome vorliegen, welche stehen im Vordergrund?	1……………………………………………………………<br>2……………………………………………………………<br>3……………………………………………………………	

Klinikum rechts der Isar Technische Universität München

CHARITÉ UNIVERSITÄTSMEDIZIN BERLIN

Munich Berlin Symptom Questionnaire (MBSQ) – Auswertungsbogen für Erwachsene (≥ 18 Jahre) bei Verdacht auf ME/CFS

Name:	Vorname:
Geburtsdatum:	
Ausfülldatum:	

Name (Arzt*in):
Datum (Arzt*in):
Einrichtung:

Nur wenn nach ärztlicher Beurteilung Häufigkeit **und** Schweregrad mit ≥**2** angegeben werden, geht der jeweilige Punkt positiv in die Auswertung ein.
ME/CFS ist eine Ausschlussdiagnose, die im Falle neuer klinischer Aspekte reevaluiert werden muss.

	Kanadische Konsensus-Kriterien (CCC)[1]		Kriterien des Institute of Medicine (IOM)[2]
O	**Krankheitsdauer (Tag des Krankheitsbeginns: __.__.____)**<br>Die Symptomatik besteht seit mindestens **6 Monaten**.	O	**Krankheitsdauer (Tag des Krankheitsbeginns: __.__.____)**<br>Die Symptomatik besteht seit mindestens **6 Monaten.**
O	**Anamnese, körperliche Untersuchung und Differentialdiagnostik**<br>☐ ergaben keinen Anhalt für eine andere Ursache der Beschwerden (insbes. kein Anhalt *für M. Addison, M. Cushing, Hypo-/ Hyperthyreose, Anämie, Hämochromatose, Diabetes mellitus, hämatoonkologische, rheumatologische und behandelbare Schlaferkrankungen*)<br>☐ Die Symptome bestehen erst seit Erkrankungsbeginn oder haben in Häufigkeit und Schwere zugenommen.	O	**Anamnese, körperliche Untersuchung und Differentialdiagnostik**<br>☐ ergaben keinen Anhalt für eine andere Ursache der Beschwerden<br>☐ Die Symptome bestehen erst seit Erkrankungsbeginn oder haben in Häufigkeit und Schwere zugenommen.
O	**Fatigue**<br>alle nachfolgenden Punkte müssen erfüllt sein:<br>☐ Fatigue: **I.1** ≥2<br>☐ Einschränkungen: **I.2** ≥2<br>☐ Fatigue neu begonnen: **I.3** Trifft zu.	O	**Fatigue**<br>alle nachfolgenden Punkte müssen erfüllt sein:<br>☐ Fatigue: **I.1** ≥2<br>☐ Einschränkungen: **I.2** ≥2<br>☐ Fatigue neu begonnen: **I.3** Trifft zu.<br>☐ Fatigue ist nicht Folge von anhaltender, exzessiver Belastung: **I.4** Trifft zu.<br>☐ Fatigue erheblich gebessert durch Ausruhen: **I.5** Trifft nicht zu.
O	**Belastungsintoleranz**<br>alle nachfolgenden Punkte müssen erfüllt sein:<br>☐ Beschwerdezunahme nach Aktivitäten; vermind. Durchhalteverm.: alle 2 Punkte **II.6-7** ≥2<br>☐ Zustandsverschlechterung: **II.8** ≥14 Std.*	O	**Belastungsintoleranz**<br>alle 2 nachfolgenden Punkte müssen erfüllt sein: **II.6-7** ≥2

	Kanadische Konsensus-Kriterien (CCC)[1]		Kriterien des Institute of Medicine (IOM)[2]
O	**Schlaf**<br>mind. 1 der nachfolgenden 5 Punkte muss erfüllt sein: **III.11-15** ≥2	O	**Schlaf**<br>der nachfolgende Punkt muss erfüllt sein: **III.11** ≥2
O	**Schmerzen**<br>mind. 1 der nachfolgenden 3 Punkte muss erfüllt sein: **IV.16-18** ≥2		**Schmerzen**<br>*nicht in IOM-Kriterien berücksichtigt*
O	**Neurokognitive Manifestationen**<br>mind. 2 der nachfolgenden Symptomgruppen müssen erfüllt sein:<br>☐ Verwirrung: **V.19** ≥2<br>☐ Konzentration u/o Gedächtnis: 1 der 2 nachfolgenden Punkte: **V.21-22** ≥2<br>☐ Orientierung: **V.23** ≥2<br>☐ Verständnis u/o Wortfindung: 1 der 2 nachfolgenden Punkte: **V.24-25** ≥2<br>☐ Wahrnehmung und Überlastungsphänomene: 1 der 5 nachfolgenden Punkte: **V.27, V.31-34** ≥2<br>☐ Koordination und Muskulatur: 1 der 3 nachfolgenden Punkte: **V.28-30** ≥2	O	**Neurokognitive und autonome Manifestationen**<br>mind. 1 der folgenden 2 Kategorien muss erfüllt sein:<br>☐ die folgenden beiden Symptomgruppen müssen erfüllt sein:<br>☐ mind. 1 der nachfolgenden 6 Punkte muss erfüllt sein: **V.20-22, V.24-25, V.28** ≥2<br>☐ der folgende Punkt muss erfüllt sein: **V.26** Ja<br>☐ Mind. 1 der nachfolgenden 2 Punkte muss erfüllt sein: **VI.37-38** ≥2
O	**Autonome, neuroendokrine und immunologische Manifestationen**<br>mind. 2 der 3 Kategorien müssen erfüllt sein:<br>☐ mind. 1 der nachfolgenden 8 Punkte muss erfüllt sein: **VI.35-42** ≥2<br>☐ mind. 1 der nachfolgenden 4 Punkte muss erfüllt sein: **VII.43-46** ≥2<br>☐ mind. 1 der nachfolgenden 4 Punkte muss erfüllt sein: **VIII.47-50** ≥2		**Neuroendokrine und immunologische Manifestationen**<br>*nicht in IOM-Kriterien berücksichtigt*

**O Patient*in erfüllt die Kanadischen Konsensus-Kriterien für ME/CFS**<br><br>**O Patient*in erfüllt <u>nicht</u> die Kanadischen Konsensus-Kriterien für ME/CFS**	**O Patient*in erfüllt die IOM-Kriterien für ME/CFS**<br><br>**O Patient*in erfüllt <u>nicht</u> die IOM-Kriterien für ME/CFS**

[1] Carruthers BM, Jain AK, De Meirleir KL, Peterson DL, Klimas NG, Lerner, AM, Bested AC, Flor-Henry P, Joshi P, Powles ACP, Sherkey JA, van de Sande MI. Myalgic Encephalomyelitis/Chronic Fatigue Syndrome. Journal of Chronic Fatigue Syndrome 2003;11:7-115. doi: 10.1300/J092v11n01_02.

[2] Clayton EW. Beyond Myalgic Encephalomyelitis/Chronic Fatigue Syndrome: An IOM Report on Redefining an Illness. JAMA. 2015;313(11):1101–1102. doi:10.1001/jama.2015.1346

* In der Originalpublikation von Carruthers BM et al.[1] ist eine PEM-Dauer von >24 Stunden gefordert. Nach aktuellem internationalen Forschungsstand empfehlen wir jedoch die Diagnosestellung schon ab einer PEM-Dauer von ≥14 Stunden, siehe
a) Cotler J, Holtzman C, Dudun C, Jason LA. A Brief Questionnaire to Assess Post-Exertional Malaise. Diagnostics. 2018; 8(3):66. doi.org/10.3390/diagnostics8030066
b) Kedor C, Freitag H, Meyer-Arndt L, Wittke K, Hanitsch LG, Zoller T, Steinbeis F, Haffke M, Rudolf G, Heidecker B, Bobbert T, Spranger J, Volk HD, Skurk C, Konietschke F, Paul F, Behrends U, Bellmann-Strobl J, Scheibenbogen C. A prospective observational study of post-COVID-19 chronic fatigue syndrome following the first pandemic wave in Germany and biomarkers associated with symptom severity. Nat Commun. 2022;13(1):5104. doi: 10.1038/s41467-022-32507-6. Erratum in: Nat Commun. 2022;13(1):6009.

MBSQ_Fragebogen_Erwachsene_Deutsch_27.10.2022_ K. Wiehler, J. Paulick, R. Pricoco, A. Leone, D.R.B. Hattesohl, L. Fröhlich, C. Scheibenbogen, U. Behrends ©

Klinikum rechts der Isar | Technische Universität München | CHARITÉ UNIVERSITÄTSMEDIZIN BERLIN

Munich Berlin Symptom Questionnaire (MBSQ) – Ärztlicher Anamnesebogen für Kinder und Jugendliche bei Verdacht auf Myalgische Enzephalomyelitis / Chronisches Fatigue-Syndrom (ME/CFS)

Name: Vorname:	Name (Arzt*in):
Geburtsdatum:	Datum (Arzt*in):
Ausfülldatum: Ausfülldauer: min	Einrichtung:

*Für Patient*innen: Bitte fülle den Fragebogen, soweit möglich, alleine aus und lasse Dir, falls nötig, von Deinen Eltern helfen.*
*Für Ärzt*innen: Dieser Bogen stellt die Grundlage für* ***ein ärztliches Gespräch*** *dar.* ***Offene Punkte*** *oder* ***Verständnisprobleme*** *müssen im* ***ärztlichen Gespräch*** *geklärt werden. Die* ***ärztliche Beurteilung*** *erfolgt anhand des* ***Auswertungsbogens für Kinder und Jugendliche****. ME/CFS ist eine* ***klinische Diagnose****. Die Diagnosestellung erfordert zusätzlich zu dieser Erhebung eine* ***angemessene Differenzialdiagnostik****.*

		**Während der letzten 3 Monate**			
		**Liegt nicht vor**	**Häufigkeit** **1** = manchmal **2** = etwa ½ der Zeit **3** = meistens **4** = immer	**Schwere** **1** = mild **2** = moderat **3** = schwer **4** = sehr schwer	**Ärztlicher Vermerk**
**I**	**Fatigue**				
**1**	Fatigue (Erschöpfung, Abgeschlagenheit, Schlappheit, Schwäche, Mangel an Energie)	0	1 2 3 4	1 2 3 4	
**2**	Einschränkungen im Alltag – Schule / Ausbildung	0	1 2 3 4	1 2 3 4	
**3**	Einschränkungen im Alltag – Sozial (Freunde, Familie)	0	1 2 3 4	1 2 3 4	
**4**	Einschränkungen im Alltag – Persönlich (Selbst)	0	1 2 3 4	1 2 3 4	

Falls Fatigue vorliegt:			
**5**	Die Fatigue hat zu einem neuen oder bestimmten Zeitpunkt begonnen (nicht lebenslang).	O Trifft zu. O Trifft nicht zu.	
**6**	Die Fatigue ist **nicht** Folge von anhaltender, exzessiver Belastung.	O Trifft zu. O Trifft nicht zu.	
**7**	Die Fatigue bessert sich deutlich durch Ausruhen.	O Trifft zu. O Trifft nicht zu.	

**II**	**Belastungsintoleranz / belastungsinduzierte Symptomverschlechterung**				
**8**	Vermindertes geistiges oder körperliches Durchhaltevermögen	0	1 2 3 4	1 2 3 4	
**9**	Vermehrte Beschwerden nach alltäglichen Aktivitäten (körperlicher, geistiger oder emotionaler Art)	0	1 2 3 4	1 2 3 4	

Falls Belastungsintoleranz vorliegt:			
**10**	Wie lange dauert es nach alltäglichen Aktivitäten bis die vermehrten Beschwerden wieder abgeklungen sind?	O ≤ 1 Std. O 2-3 Std. O 4-10 Std.<br>O 11-13 Std. O 14-23 Std. O ≥ 24 Std.	
**11**	Welche drei alltäglichen Aktivitäten führen zu vermehrten Beschwerden?	1.............<br>2.............<br>3.............	
**12**	Welche drei Symptome verschlechtern sich nach alltäglichen Aktivitäten?	1.............<br>2.............<br>3.............	

**III**	**Schlaf**				
**13**	Unerholsamer Schlaf	0	1 2 3 4	1 2 3 4	
**14**	Ungewöhnlich viel Schlaf tagsüber	0	1 2 3 4	1 2 3 4	
**15**	Einschlafprobleme	0	1 2 3 4	1 2 3 4	
**16**	Durchschlafprobleme	0	1 2 3 4	1 2 3 4	
**17**	Verschobener Tag-Nacht-Rhythmus	0	1 2 3 4	1 2 3 4	

**IV**	**Schmerzen**				
**18**	Muskelschmerzen, muskelkaterartiges Gefühl	0	1 2 3 4	1 2 3 4	
**19**	Gelenkschmerzen ohne Schwellung oder Rötung	0	1 2 3 4	1 2 3 4	
**20**	Kopfschmerzen	0	1 2 3 4	1 2 3 4	
**21**	Bauchschmerzen	0	1 2 3 4	1 2 3 4	

		Während der letzten 3 Monate		
	Liegt nicht vor	**Häufigkeit**<br>**1** = manchmal<br>**2** = etwa ½ der Zeit<br>**3** = meistens<br>**4** = immer	**Schwere**<br>**1** = mild<br>**2** = moderat<br>**3** = schwer<br>**4** = sehr schwer	**Ärztlicher Vermerk**
**V Neurokognitive Manifestationen**				
**22** Verwirrung	0	1 2 3 4	1 2 3 4	
**23** Verlangsamtes Denken	0	1 2 3 4	1 2 3 4	
**24** Konzentrationsprobleme	0	1 2 3 4	1 2 3 4	
**25** Probleme mit dem Kurzzeitgedächtnis	0	1 2 3 4	1 2 3 4	
**26** Orientierungsprobleme	0	1 2 3 4	1 2 3 4	
**27** Verständnisprobleme / Aufmerksamkeitsprobleme	0	1 2 3 4	1 2 3 4	
**28** Wortfindungsstörungen	0	1 2 3 4	1 2 3 4	

**29** Falls kognitive Beschwerden (**V.23-28**) vorliegen, verschlechtern sich diese durch Anstrengung, anhaltende aufrechte Position, Stress oder Zeitdruck?	Ja Nein	

**30** Geistesabwesenheit	0	1 2 3 4	1 2 3 4	
**31** Schwierigkeiten, Informationen abzurufen	0	1 2 3 4	1 2 3 4	
**32** Mangelnde Multitaskingfähigkeit	0	1 2 3 4	1 2 3 4	
**33** Schwierigkeiten beim Ausdrücken von Gedanken	0	1 2 3 4	1 2 3 4	
**34** Beim Denken oft den Faden verlieren	0	1 2 3 4	1 2 3 4	
**35** Neue Schulprobleme (z.B. in Mathematik)	0	1 2 3 4	1 2 3 4	
**36** Wahrnehmungs-, Empfindungs- und/oder Sinnesstörungen (z.B. verschwommenes Sehen)	0	1 2 3 4	1 2 3 4	
**37** Koordinationsschwierigkeiten	0	1 2 3 4	1 2 3 4	
**38** Muskelzuckungen	0	1 2 3 4	1 2 3 4	
**39** Muskelschwäche	0	1 2 3 4	1 2 3 4	
**40** Lichtempfindlichkeit	0	1 2 3 4	1 2 3 4	
**41** Geräuschempfindlichkeit	0	1 2 3 4	1 2 3 4	
**42** Berührungsempfindlichkeit	0	1 2 3 4	1 2 3 4	
**43** Überlastung durch positive und/oder negative Emotionen	0	1 2 3 4	1 2 3 4	

**VI Autonome Manifestationen**				
**44** Schwindel / Benommenheit	0	1 2 3 4	1 2 3 4	
**45** Herzklopfen / Herzrasen	0	1 2 3 4	1 2 3 4	
**46** Kreislaufprobleme beim Aufrichten	0	1 2 3 4	1 2 3 4	
**47** Kreislaufprobleme in aufrechter Position	0	1 2 3 4	1 2 3 4	
**48** Ungewohnte Blässe	0	1 2 3 4	1 2 3 4	
**49** Blasenprobleme	0	1 2 3 4	1 2 3 4	
**50** Magen-Darm-Probleme	0	1 2 3 4	1 2 3 4	
**51** Belastungsabhängige Atembeschwerden	0	1 2 3 4	1 2 3 4	

**VII Neuroendokrine Manifestationen**				
**52** Temperaturregulationsstörung (z.B. erniedrigte Temperatur, Temperaturschwankungen, Schwitzen, fiebriges Gefühl, kalte Hände/ Füße)	0	1 2 3 4	1 2 3 4	
**53** Unverträglichkeit von extremer Hitze / Kälte	0	1 2 3 4	1 2 3 4	
**54** Gewichtsveränderung bei Appetitveränderung	0	1 2 3 4	1 2 3 4	
**55** Symptomverschlechterung bei Stress	0	1 2 3 4	1 2 3 4	

**VIII Immunologische Manifestationen**				
**56** Grippiges Gefühl / generelles Krankheitsgefühl	0	1 2 3 4	1 2 3 4	
**57** Schmerzhafte/ empfindliche Lymphknoten	0	1 2 3 4	1 2 3 4	
**58** Fieber	0	1 2 3 4	1 2 3 4	
**59** Wiederkehrende Halsschmerzen	0	1 2 3 4	1 2 3 4	
**60** Neue Empfindlichkeit gegenüber Lebensmitteln, Medikamenten, Chemikalien, etc.	0	1 2 3 4	1 2 3 4	

**Sonstige Fragen zum Symptomverlauf**		
Falls bei Dir mehr als drei der genannten Symptome vorliegen, welche stehen im Vordergrund?	1.……………………………………<br>2.……………………………………<br>3.……………………………………	

Klinikum rechts der Isar Technische Universität München

CHARITÉ UNIVERSITÄTSMEDIZIN BERLIN

Munich Berlin Symptom Questionnaire (MBSQ) – Auswertungsbogen Nr. 1 für Kinder und Jugendliche (0-17 Jahre) bei Verdacht auf ME/CFS

Name: Vorname:	Name (Arzt*in):
Geburtsdatum:	Datum (Arzt*in):
Ausfülldatum:	Einrichtung:

Nur wenn nach ärztlicher Beurteilung Häufigkeit **und** Schweregrad mit ≥**2** angegeben werden, geht der jeweilige Punkt positiv in die Auswertung ein.
ME/CFS ist eine Ausschlussdiagnose, die im Falle neuer klinischer Aspekte reevaluiert werden muss.

	Pädiatrische Falldefinition für ME/CFS nach Jason LA et al.[1]		Klinisch-diagnostisches Arbeitsblatt nach Rowe PC et al.[2]
O	**Krankheitsdauer (Tag des Krankheitsbeginns: __.__.____)**<br>☐ Die Symptomatik besteht seit mindestens **3 Monaten**.	O	**Krankheitsdauer (Tag des Krankheitsbeginns: __.__.____)**<br>☐ Die Symptomatik besteht seit mindestens **3 Monaten.***
O	**Anamnese, körperliche Untersuchung und Differentialdiagnostik**<br>ergaben keinen Anhalt für eine andere Ursache der Beschwerden<br>(insbesondere kein Anhalt für *unbehandelte Hypothyreose, Schlafapnoe, Narkolepsie, maligne Erkrankungen, Leukämie, Hepatitis, Multiple Sklerose, Juvenile rheumatoide Arthritis, systemischer Lupus erythematodes, HIV/AIDS, schwere Adipositas (BMI>40), Zöliakie, Borreliose, kindliche Schizophrenie oder psychotische Störungen, Bipolare Störung, Aktiver Alkohol oder –Substanzmissbrauch, aktive Anorexie oder Bulimie, Depression)*	O	**Anamnese, körperliche Untersuchung und Differentialdiagnostik**<br>ergaben keinen Anhalt für eine andere Ursache der Beschwerden<br>(insbesondere kein Anhalt für *NNR-Insuffizienz, Übertrainingssyndrom, GI-Störungen: Zöliakie, Reizdarmsyndrom, eosinophile Gastroenteritis, Chiari Malformation, zervikale Spinalkanalstenose, Neuroborreliose oder andere durch Zecken übertragbare Erkrankungen, Depression, Narkolepsie, obstruktive oder zentrale Schlafapnoe, post-kommotionelles Syndrom, schwere Anämie, systemischer Lupus erythematodes und ähnliche Autoimmunerkrankungen, unbehandelte Hypo-/ Hyperthyreose)*
O	**Fatigue**<br>alle nachfolgenden Punkte müssen erfüllt sein:<br>☐ Fatigue: **I.1** ≥2<br>☐ Einschränkungen: alle 3 Punkte: **I.2-4** ≥2<br>☐ Fatigue ist nicht Folge von anhaltender, exzessiver Belastung: **I.6** Trifft zu.<br>☐ Fatigue erheblich gebessert durch Ausruhen: **I.7** Trifft nicht zu.	O	**Fatigue**<br>alle nachfolgenden Punkte müssen erfüllt sein:<br>☐ Fatigue: **I.1** ≥2<br>☐ Einschränkungen: mind. 1 der 3 Punkte: **I.2-4** ≥2<br>☐ Fatigue ist nicht Folge von anhaltender, exzessiver Belastung: **I.6** Trifft zu.<br>☐ Fatigue erheblich gebessert durch Ausruhen: **I.7** Trifft nicht zu.
O	**Belastungsintoleranz**<br>alle nachfolgenden Punkte müssen erfüllt sein:<br>☐ Beschwerdezunahme nach Aktivitäten; vermind. Durchhalteverm.: alle 2 Punkte **II.8-9** ≥2<br>☐ Zustandsverschlechterung: **II.10** ≥14 Std.	O	**Belastungsintoleranz**<br>alle nachfolgenden Punkte müssen erfüllt sein:<br>☐ Beschwerdezunahme nach Aktivitäten; vermind. Durchhalteverm.: alle 2 Punkte: **II.8-9** ≥2<br>☐ Zustandsverschlechterung: **II.10** >24 Std.

**Pädiatrische Falldefinition für ME/CFS nach Jason LA et al.**[1]		**Klinisch-diagnostisches Arbeitsblatt nach Rowe PC et al.**[2]	
O	**Schlaf**<br>mind. 1 der nachfolgenden 5 Punkte muss erfüllt sein: **III.13-17** ≥2	O	**Schlaf, Schmerz und neurokognitive Symptome**<br>mind. 2 der 3 Kategorien müssen erfüllt sein:<br>☐ mind. 1 der nachfolgenden 5 Punkte muss erfüllt sein: **III.13-17** ≥2<br>☐ mind. 1 der nachfolgenden 6 Punkte muss erfüllt sein: **IV.18-21, VIII.57, VIII.59** ≥2<br>☐ mind. 1 der nachfolgenden 8 Punkte muss erfüllt sein: **V.23-25, V.27-28, V.30, V.33** ≥2; **V.29**: Ja
O	**Schmerzen**<br>mind. 1 der nachfolgenden 4 Punkte muss erfüllt sein: **IV.18-21** ≥2		
O	**Neurokognitive Manifestationen**<br>mind. 2 der nachfolgenden 11 Punkte müssen erfüllt sein: **V.23-25, V.27-28, V.30-35** ≥2		
O	**Autonome, neuroendokrine und immunologische Manifestationen**<br>mind. 2 der 3 Kategorien müssen erfüllt sein:<br>☐ mind. 1 der nachfolgenden 5 Punkte muss erfüllt sein: **VI.44-47, VI.51** ≥2<br>☐ mind. 1 der nachfolgenden 4 Punkte muss erfüllt sein: **VII.52-55** ≥2<br>☐ mind. 1 der nachfolgenden 4 Punkte muss erfüllt sein: **VIII.56-59** ≥2		**Autonome, neuroendokrine und immunologische Manifestationen**<br>*nicht in Diagnosekriterien nach Rowe PC et al. berücksichtigt*

O **Patient*in erfüllt die Kriterien für ME/CFS nach Jason LA et al.**	O **Patient*in erfüllt die Kriterien für ME/CFS nach Rowe PC et al.**
O **Patient*in erfüllt <u>nicht</u> die Kriterien für ME/CFS nach Jason LA et al.**	O **Patient*in erfüllt <u>nicht</u> die Kriterien für ME/CFS nach Rowe PC et al.**

[1]Jason LA, Jordan K, Miike T, Bell DS, Lapp C, Torres-Harding S, Rowe K, Gurwitt A, De Meirleir K, Van Hoof ELS. Pediatric Case Definition for Myalgic Encephalomyelitis and Chronic Fatigue Syndrome. Journal of Chronic Fatigue Syndrome 2006; 13:1-44. doi: 10.1300/J092v13n02_01.

[2] Rowe PC, Underhill RA, Friedman KJ, Gurwitt A, Medow MS, Schwartz MS, Speight N, Stewart JM, Vallings R, Rowe KS. Myalgic Encephalomyelitis/Chronic Fatigue Syndrome Diagnosis and Management in Young People: A Primer. Front Pediatr. 2017;5:121. doi: 10.3389/fped.2017.00121.

* In der Originalpublikation von Rowe PC et al.[2] ist eine Krankheitsdauer von mind. 6 Monaten für die Diagnosestellung gefordert. Bei einer Dauer <6 Monate wird dort nur empfohlen, den dringenden Verdacht zu äußern. Wir empfehlen bei Kindern und Jugendlichen bereits eine Diagnosestellung nach einer Krankheitsdauer von 3 Monaten, um sie frühzeitig symptomorientiert zu versorgen.

Klinikum rechts der Isar Technische Universität München

CHARITÉ UNIVERSITÄTSMEDIZIN BERLIN

Munich Berlin Symptom Questionnaire (MBSQ) – Auswertungsbogen Nr. 2 für Kinder und Jugendliche (0-17 Jahre) bei Verdacht auf ME/CFS

Name: Vorname:	Name (Arzt*in):
Geburtsdatum:	Datum (Arzt*in):
Ausfülldatum:	Einrichtung:

Nur wenn Häufigkeit **und** Schweregrad mit ≥**2** angegeben werden, geht der jeweilige Punkt positiv in die Auswertung ein.
ME/CFS ist eine Ausschlussdiagnose, die im Falle neuer klinischer Aspekte reevaluiert werden muss.

	Kanadische Konsensus-Kriterien (CCC)[3]		Kriterien des Institute of Medicine (IOM)[4]
O	**Krankheitsdauer (Tag des Krankheitsbeginns: __.__.____)**<br>Die Symptomatik besteht seit mindestens **3 Monaten**.	O	**Krankheitsdauer (Tag des Krankheitsbeginns: __.__.____)**<br>Die Symptomatik besteht seit mindestens **3 Monaten**.**
O	**Anamnese, körperliche Untersuchung und Differentialdiagnostik**<br>☐ ergaben keinen Anhalt für eine andere Ursache der Beschwerden (insbes. kein Anhalt für *M. Addison, M. Cushing, Hypo-/ Hyperthyreose, Anämie, Hämochromatose, Diabetes mellitus, hämatoonkologische, rheumatologische und behandelbare Schlaferkrankungen*)<br>☐ Die Symptome bestehen erst seit Erkrankungsbeginn oder haben in Häufigkeit und Schwere zugenommen.	O	**Anamnese, körperliche Untersuchung und Differentialdiagnostik**<br>☐ ergaben keinen Anhalt für eine andere Ursache der Beschwerden.<br>☐ Die Symptome bestehen erst seit Erkrankungsbeginn oder haben in Häufigkeit und Schwere zugenommen.
O	**Fatigue**<br>alle nachfolgenden Punkte müssen erfüllt sein:<br>☐ Fatigue: **I.1** ≥2<br>☐ Einschränkungen: mind. 1 von 3 Punkten: **I.2-4** ≥2<br>☐ Fatigue neu begonnen: **I.5** Trifft zu.	O	**Fatigue**<br>alle nachfolgenden Punkte müssen erfüllt sein:<br>☐ Fatigue: **I.1** ≥2<br>☐ Einschränkungen: mind. 1 von 3 Punkten: **I.2-4** ≥2<br>☐ Fatigue neu begonnen: **I.5** Trifft zu.<br>☐ Fatigue ist nicht Folge von anhaltender, exzessiver Belastung: **I.6** Trifft zu.<br>☐ Fatigue deutlich gebessert durch Ausruhen: **I.7** Trifft nicht zu.
O	**Belastungsintoleranz**<br>alle nachfolgenden Punkte müssen erfüllt sein:<br>☐ Beschwerdezunahme nach Aktivitäten; vermind. Durchhalteverm.: alle 2 Punkte **II.8-9** ≥2<br>☐ Zustandsverschlechterung: **II.10** ≥14 Std.*	O	**Belastungsintoleranz**<br>alle 2 nachfolgenden Punkte müssen erfüllt sein: **II.8-9** ≥2

	Kanadische Konsensus-Kriterien (CCC)[3]		Kriterien des Institute of Medicine (IOM)[4]
O	**Schlaf**<br>mind. 1 der nachfolgenden 5 Punkte muss erfüllt sein: **III.13-17** ≥2	O	**Schlaf**<br>der nachfolgende Punkt muss erfüllt sein: **III.13** ≥2
O	**Schmerzen**<br>mind. 1 der nachfolgenden 3 Punkte muss erfüllt sein: **IV.18-20** ≥2<br>*(ausgenommen Bauchschmerzen)*		**Schmerzen**<br>*nicht in IOM-Kriterien berücksichtigt*
O	**Neurokognitive Manifestationen**<br>mind. 2 der nachfolgenden Symptomgruppen müssen erfüllt sein:<br>☐ Verwirrung: **V.22** ≥2<br>☐ Konzentration u/o Gedächtnis: 1 der 2 nachfolgenden Punkte: **V.24-25** ≥2<br>☐ Orientierung: **V.26** ≥2<br>☐ Verständnis u/o Wortfindung: 1 der 2 nachfolgenden Punkte: **V.27-28** ≥2<br>☐ Wahrnehmung und Überlastungsphänomene: 1 der 5 nachfolgenden Punkte: **V.36, V.40-43** ≥2<br>☐ Koordination und Muskulatur: 1 der 3 nachfolgenden Punkte: **V.37-39** ≥2	O	**Neurokognitive und autonome Manifestationen**<br>mind. 1 der folgenden 2 Kategorien muss erfüllt sein:<br>☐ die folgenden beiden Symptomgruppen müssen erfüllt sein:<br>☐ mind. 1 der nachfolgenden 6 Punkte muss erfüllt sein: **V.23-25, V.27-28, V.37** ≥2<br>☐ der folgende Punkt muss erfüllt sein: **V.29** Ja<br>☐ Mind. 1 der nachfolgenden 2 Punkte muss erfüllt sein: **VI.46-47** ≥2
O	**Autonome, neuroendokrine und immunologische Manifestationen**<br>mind. 2 der 3 Kategorien müssen erfüllt sein:<br>☐ mind. 1 der nachfolgenden 8 Punkte muss erfüllt sein: **VI.44-51** ≥2<br>☐ mind. 1 der nachfolgenden 4 Punkte muss erfüllt sein: **VII.52-55** ≥2<br>☐ mind. 1 der nachfolgenden 4 Punkte muss erfüllt sein: **VIII.56-57, VIII.59-60** ≥2		**Neuroendokrine und immunologische Manifestationen**<br>*nicht in IOM-Kriterien berücksichtigt*

**O Patient*in erfüllt die Kanadischen Konsensus-Kriterien für ME/CFS**	**O Patient*in erfüllt die IOM-Kriterien für ME/CFS**
**O Patient*in erfüllt die Kanadischen Konsensus-Kriterien für ME/CFS <u>nicht</u>**	**O Patient*in erfüllt die IOM-Kriterien für ME/CFS <u>nicht</u>**

[3] Carruthers BM, Jain AK, De Meirleir KL, Peterson DL, Klimas NG, Lerner, AM, Bested AC, Flor-Henry P, Joshi P, Powles ACP, Sherkey JA, van de Sande MI. Myalgic Encephalomyelitis/Chronic Fatigue Syndrome. Journal of Chronic Fatigue Syndrome 2003;11:7-115. doi: 10.1300/J092v11n01_02.

[4] Clayton EW. Beyond Myalgic Encephalomyelitis/Chronic Fatigue Syndrome: An IOM Report on Redefining an Illness. JAMA. 2015;313(11):1101–1102. doi:10.1001/jama.2015.1346.

* In der Originalpublikation von Carruthers BM et al.[3] ist eine PEM-Dauer von >24 Stunden gefordert. Nach aktuellem internationalen Forschungsstand empfehlen wir jedoch die Diagnosestellung schon ab einer PEM-Dauer von ≥14 Stunden. siehe a) Cotler J et al. Diagnostics. 2018;8(3):66. doi.org/10.3390/diagnostics8030066 und b) Kedor C et al., Nat Commun. 2022;13(1):5104. doi: 10.1038/s41467-022-32507-6. Erratum in: Nat Commun. 2022;13(1):6009.

** In der Originalpublikation von Clayton EW et al.[4] ist eine Krankheitsdauer von mind. 6 Monaten für die Diagnosestellung gefordert. Wir empfehlen bei Kindern und Jugendlichen bereits eine Diagnosestellung nach einer Krankheitsdauer von 3 Monaten, um sie frühzeitig symptomorientiert zu versorgen.

MBSQ_supplementary scoring sheet_adults_English_2021 07 29_K. Wiehler, J. Paulick, R. Pricoco, A. Leone, D.R.B. Hattesohl, L. Fröhlich, C. Scheibenbogen, U. Behrends ©
